# Disparities in the unmet mental health needs between LGBTQ+ and non-LGBTQ+ populations during COVID-19 in the United States from 21 July 2021 to 9 May 2022

**DOI:** 10.3389/fmed.2022.995466

**Published:** 2022-11-08

**Authors:** Shanquan Chen, Yuqi Wang, Rui She, Pei Qin, Wai-Kit Ming

**Affiliations:** ^1^Department of Psychiatry, University of Cambridge, Cambridge, United Kingdom; ^2^Department of Mathematics, Imperial College London, London, United Kingdom; ^3^Department of Computer Science, University College London, London, United Kingdom; ^4^Department of Rehabilitation Sciences, The Hong Kong Polytechnic University, Shatin, Hong Kong SAR, China; ^5^Shenzhen Qianhai Shekou Free Trade Zone Hospital, Shenzhen, China; ^6^School of Public Health, Shenzhen University Health Science Center, Shenzhen, China; ^7^Massachusetts General Hospital and Harvard Medical School, Harvard University, Boston, MA, United States; ^8^Department of Infectious Diseases and Public Health, Jockey Club College of Veterinary Medicine and Life Sciences, City University of Hong Kong, Kowloon, Hong Kong SAR, China

**Keywords:** unmet mental health needs, LGBTQ+, COVID-19, US, disparity

## Abstract

**Background:**

Evidence highlighted the likelihood of unmet mental health needs (UMHNs) among LGBTQ+ than non-LGBTQ+ populations during COVID-19. However, there lacks evidence to accurately answer to what extent the gap was in UMHN between LGBTQ+ and non-LGBTQ+ populations. We aim to evaluate the difference in UMHN between LGBTQ+ and non-LGBTQ+ during COVID-19.

**Methods:**

Cross-sectional data from Household Pulse Survey between 21 July 2021 and 9 May 2022 were analyzed. LGBTQ+ was defined based on self-reported sex at birth, gender, and sexual orientation identity. UMHN was assessed by a self-reported question. Multivariable logistic regressions generated adjusted odds ratios (AODs) of UMHN, both on overall and subgroups, controlling for a variety of socio-demographic and economic-affordability confounders.

**Findings:**

81267 LGBTQ+ and 722638 non-LGBTQ+ were studied. The difference in UMHN between LGBTQ+ and non-LGBTQ+ (as reference) varied from 4.9% (95% CI 1.2–8.7%) in Hawaii to 16.0% (95% CI 12.2–19.7%) in Utah. In multivariable models, compared with non-LGBTQ+ populations, LGBTQ+ had a higher likelihood to report UMHN (AOR = 2.27, 95% CI 2.18–2.39), with the highest likelihood identified in transgender (AOR = 3.63, 95% CI 2.97–4.39); compared with LGBTQ+ aged 65+, LGBTQ+ aged 18–25 had a higher likelihood to report UMHN (AOR = 1.34, 95% CI 1.03–1.75); compared with White LGBTQ+ populations, Black and Hispanic LGBTQ+ had a lower likelihood to report UMHN (AOR = 0.72, 95% CI 0.63–0.82; AOR = 0.85, 95% CI 0.75–0.97, respectively).

**Interpretation:**

During the COVID-19, LGBTQ+ had a substantial additional risk of UMHN than non-LGBTQ+. Disparities among age groups, subtypes of LGBTQ+, and geographic variance were also identified.

## Introduction

The COVID-19 crisis has disrupted the mental health services when such services are needed more than ever, with marginalized members of society disproportionately influenced (1, 2). A rising worry argued that most of our attention had been placed on visible marginalized groups (like ethnic minorities). However, less visible groups, such as people from communities of lesbian, gay, bisexual, transgender, queer, and other people of diverse sexual orientation and gender identity (LGBTQ+), have received relatively less attention (3–5).

Prior to the emergence of COVID-19, LGBTQ+ populations had experienced greater mental health problems and unmet mental health needs (UMHN) than non-LGBTQ+ populations, because of stigma, discrimination, economic vulnerabilities, and less availability of identity-affirming services (5–10). The COVID-19 crisis has led to negative impacts on the lives of many, but its effects are further exacerbating the aforementioned existing risks and barriers among LGBTQ+ populations. A survey based on 1,000 adults in the United States found that during the COVID-19 crisis, about 40% of LGBTQ+ households experienced barriers to medical care, compared with 19% of non-LGBTQ+ households (11). Similar findings were identified from other groups of vulnerable populations and countries. Studies from the United States indicated that people with disabilities experienced significant delays in medical care, because of severe disruptions in access to accessible transportation (12), non-emergency medical services (13), and personal assistance services and home healthcare (14, 15). A study based on 26 countries in Europe indicated that unmet healthcare needs were primarily induced by having pre-scheduled care postponed (accounting for 25%), forgoing care for fear of contracting COVID-19 (accounting for 12%), and being unable to obtain medical appointments or treatments when needed (accounting for 5%) (16). Extensive literature indicated that the COVID-19 pandemic has increased mental health needs (17, 18). Superimposed with the aforementioned barriers also incurred by COVID-19, the UMHN could be worse.

The COVID-19 crisis also had impacts on the LGBTQ+ community in a unique manner. There have been documented cancelations and delays in gender-affirming surgeries, which were associated with negative mental health consequences (2). Furthermore, LGBTQ+ with intersecting marginalized identities (like LGBTQ+ people of color and young LGBTQ+) could be more vulnerable during the COVID-19 crisis. A survey based on 4,000 adults in the United States found that during the COVID-19 crisis, 22% of LGBTQ+ people of color became unemployed, compared to 14% of white LGBTQ+ people and 13% of the general population (19). The shutdown of schools or universities that could provide a gateway to mental health services further compounded the mental health burden in young LGBTQ+ individuals (5).

The available evidence highlighted the substantial additional likelihood of UMHN among LGBTQ+ than non-LGBTQ+ populations. However, there is still a lack of evidence to accurately answer: during COVID-19, to what extent the mental health needs of LGBTQ+ were met or to what extent the gap was in UMHN between LGBTQ+ and non-LGBTQ+ populations. In this study, we evaluated the UMHN gap between LGBTQ+ and non-LGBTQ+ populations, with focuses on age and race/ethnicity disparities.

## Materials and methods

### Data source and participants

Data are from the Household Pulse Survey (HPS), which is a nationally representative survey of adults (age = 18) measuring the impact of the COVID-19 pandemic and was conducted by the US Census Bureau in partnership with the Centers for Disease Control and Prevention (20). The HPS used the US Census Bureau’s Master Address File as the source of sampled housing units. The sample design was a systematic sample of all eligible housing units, with adjustments applied to the sampling intervals to select a large enough sample to create representative estimates at the national, state, and metropolitan area levels. Technical details are available on the Census Bureau website (21). HPS was administered online and collected information on demographic, socioeconomic, and health status. We utilized HPS data spanning 21 July 2021 through 9 May 2022, as this period has data collected on both UMHN and gender identity and sexual orientation identity.

The data are publicly available. The use of secondary de-identified data makes this study exempt from institutional review board review. This study follows the Strengthening the Reporting of Observational Studies in Epidemiology (STROBE) reporting guideline (22).

### Outcome and measures

Unmet mental health needs was assessed by the question “At any time in the last 4 weeks, did you need counseling or therapy from a mental health professional, but did not get it for any reason,” with the response yes or no.

LGBTQ+ status was defined by self-reported sex at birth (male or female), gender identity (male, female, transgender, or none of these), and sexual orientation identity (gay/lesbian, straight, bisexual, something else, or don’t know). Non-LGBTQ+ populations were defined as those who are heterosexual and have the same birth sex and gender identity. The remaining populations were grouped as LGBTQ+. Subtypes of LGBTQ+ were also separated: Lesbian was defined as those who had the female birth sex and had the answer “gay/lesbian” for sexual orientation; gay was defined as those who had the male birth sex and had the answer “gay/lesbian” for sexual orientation; bisexual was defined as those who had bisexual sexual orientation; transgender was defined as those who had transgender gender identity; remaining populations from LGBTQ+ was categorized into queer and other people of diverse sexual orientation and gender identity (queer+).

#### Covariates

We examined the following socio-demographic variables: age (18–25, 26–49, 50–64, vs. 65+), race/ethnicity (White, Black, Hispanic, vs. Asian and others), marital status (married, cohabiting or civil partnership, never married, vs. widowed/divorced/separated), and education attained (less than high school, some high school, high school graduate or equivalent, some college but degree not received or is in progress, associate’s degree, bachelor’s degree, vs. graduate degree). We also investigated the affordability of mental health service, reflected by total household income before taxes ($0–$34,999, $35,000–$49,999, $50,000–$74,999, $75,000–$99,999, vs. $100,000 +), difficulty with expenses (yes or no), availability of public health insurance (yes or no), and availability of private health insurance (yes or no). Difficulty with expenses was assessed by the question “In the last 7 days, how difficult has it been for your household to pay for usual household expenses, including but not limited to food, rent or mortgage, car payments, medical expenses, student loans, and so on?” with the response not at all difficult, a little difficult, somewhat difficult, and very difficult. Responses with somewhat difficult and very difficult were recorded as “Yes.” People were regarded as “has public insurance” if they had any one of the following types of insurance: (a) Medicare; (b) Medicaid, Medical Assistance, or any kind of government-assistance plan for those with low incomes or a disability; (c) VA (including those who have ever used or enrolled for VA healthcare). People were regarded as “has private insurance” if they had any one of the following types of insurance: (a) Insurance through a current or former employer or union (through yourself or another family member); (b) Insurance purchased directly from an insurance company, including marketplace coverage (through yourself or another family member); (c) TRICARE or other military healthcare.

#### State-level measures

We extracted state-level measures in the United States from the National Mental Health Services Survey (N-MHSS). N-MHSS is a survey that collects data on the services and characteristics of all known mental health treatment facilities in the 50 states, the District of Columbia, and the U.S. territories and jurisdictions and is the only source of state-level data on the mental health service delivery system reported by both public and private specialty mental health treatment facilities (23). The data collected by N-MHSS included but were not limited to the following: what treatment was offered in the facility, how the treatment was offered, what kinds of age groups were targeted, what types of payments were accepted, and whether the facility was issued a license/certification. We extracted 131 state-level measures collected by N-MHSSS in 2020. The full list of these 131 state-level measures and their explanation can be found in the Supplementary Table 1. Following the suggestion of a previous study (24), state-level densities of corresponding measures were calculated as the proportion of each state’s total number of facilities (taking facility offering mental health diagnostic evaluation as an example, we calculated the proportion of facility which offers mental health diagnostic evaluation out of the total number of facilities in each state).

### Statistical analysis

Descriptive statistics (counts and percentage) were reported by the LGBTQ+ status (yes vs. no) and were tested by chi-squared tests.

Unmet mental health needs between LGBTQ+ and non-LGBTQ+ populations were compared using logistic regression models with LGBTQ+ (yes vs. no) as the key predictor. The results were reported as both unadjusted and adjusted odds ratios (AORs). For adjusted ORs, the controlled covariates included socio-demographics (age, race/ethnicity, marital status, and educational attainment), affordability (household income, difficulty with expenses, availability of public health insurance, and availability of private health insurance), and state of residence. Survey weights were used to account for sampling design (including the unequal probability of selection, clustering, and stratification) and generate representative estimates. The weight values were provided directly in the HRS datasets. The details of how the weights were calculated can be found elsewhere (25). Multicollinearity was tested using variance inflation factor (VIF). VIF = 10 indicates a sign of severe or serious multicollinearity (26). In this study, all models have a maximum VIF of 1.31 suggesting a negligible amount of multicollinearity.

To estimate age and race/ethnicity disparities, we also fitted similar weighted multivariable logistic regressions but added an interaction between the interested subgroup factor and LGBTQ+ status.

To show the geographic variation in the difference of UMHN between LGBTQ+ and non-LGBTQ+, we repeated the above weighted multivariable logistic regression for each state, controlling for the same socio-demographic and affordability factors. The results from the state-specific logistic regressions were used to appropriately estimate the adjusted risk difference (ARDs). The ARDs were used to graphically show the geographic variation in the difference of UMHN between LGBTQ+ and non-LGBTQ+.

We explored further the association of the above adjusted risk difference (ARD) with 131 state-level factors relating to the characteristics of mental health facilities. We used linear regression with the ARDs as the outcome and these country-level factors as predictors. Because there is a strong correlation between state-level variables, and the number of state-level variables to be fitted is much larger than the number of states, we only include one variable at a time when exploring state-level associations.

All the above analyses were repeated by subtype of LGBTQ+.

All analyses were performed using R, version 3.6.0. We report two-sided *P*-values and 95% CIs throughout. *P* < 0.05 was considered to be statistically significant.

## Results

81,267 LGBTQ+ and 722,638 non-LGBTQ+ were interviewed across 51 states. Compared with non-LGBTQ+, LGBTQ+ populations were more likely to be younger (*p* < 0.001), to be never married (*p* < 0.001), to be non-White (*p* < 0.001), to have low education (*p* < 0.001), to have low household income (*p* < 0.001), to have difficulty with expenses (*p* < 0.001), to have no public insurance (*p* < 0.001), and to have no private insurance (*p* < 0.001) (Table 1).

**TABLE 1:** Characteristics of participants in the United States from 21 July 2021 to 9 May 2022.

	Non-LGBTQ+ (*N* = 722,638)^a^	LGBTQ+ (*N* = 81,267)^a^	*P*
**Outcome**			
Unmet mental health needs (=Yes)	58586 (8.1%)	16135 (19.9%)	<0.001
**Covariates**			
**Age**			
18–25	19536 (2.7%)	8678 (10.7%)	< 0.001
26–49	264544 (36.6%)	41087 (50.6%)	
50–64	220340 (30.5%)	19002 (23.4%)	
65+	218218 (30.2%)	12500 (15.4%)	
**Race/ethnicity**			
White	541228 (74.9%)	56683 (69.7%)	< 0.001
Black	55782 (7.7%)	5422 (6.7%)	
Hispanic	64607 (8.9%)	10718 (13.2%)	
Asian and others	61021 (8.4%)	8444 (10.4%)	
**Marital status**			
Married	430889 (59.6%)	29446 (36.2%)	<0.001
Never married	119781 (16.6%)	36653 (45.1%)	
Widowed/divorced /separated	171968 (23.8%)	15168 (18.7%)	
**Total household income before taxes**			
0–$34999	139668 (19.3%)	23408 (28.8%)	<0.001
35,000–$49,999	77518 (10.7%)	10073 (12.4%)	
50,000–$74,999	123144 (17%)	13821 (17%)	
75,000–$99,999	103687 (14.3%)	10388 (12.8%)	
100,000+	278621 (38.6%)	23577 (29%)	
**Education attained**			
Less than high school	4133 (0.6%)	1256 (1.5%)	< 0.001
Some high school	9645 (1.3%)	1667 (2.1%)	
High school graduate or equivalent	85609 (11.8%)	8651 (10.6%)	
Some college, but degree not received or is in progress	150482 (20.8%)	18842 (23.2%)	
Associate’s degree	76669 (10.6%)	7485 (9.2%)	
Bachelor’s degree	207606 (28.7%)	23167 (28.5%)	
Graduate degree	188494 (26.1%)	20199 (24.9%)	
Difficulty with expenses (=Yes)	156256 (21.6%)	25387 (31.2%)	<0.001
Has public health insurance (=Yes)	253914 (35.1%)	22299 (27.4%)	<0.001
Has private health insurance (=Yes)	489758 (67.8%)	52295 (64.3%)	<0.001

Data were presented as number (percentage). *P*-values were extracted from chi-square tests.

The overall weighted prevalence of UMHN among LGBTQ+ was 20.0% (95% CI 19.5–20.6%), significantly higher than that among non-LGBTQ+ (7.8% [95% CI 7.7–8.0%]) (*p* < 0.001). After adjusting for socio-demographics and affordability, compared with non-LGBTQ+ populations, LGBTQ+ had 2.27 times likelihood to report UMHN (AOR = 2.27, 95% CI 2.18–2.39) (Table 2, model 3). Specifically, this likelihood was 2.27 times among lesbian (AOR = 2.27, 95% CI 2.01–2.59), 1.75 times among gay (AOR = 1.75, 95% CI 1.51–2.03), 2.80 times among bisexual (AOR = 2.80, 95% CI 2.69–2.92), 3.63 times among transgender (AOR = 3.63, 95% CI 2.97–4.39), and 1.99 times among queer+ (AOR = 1.99, 95% CI 1.84–2.16) (Supplementary Table 2).

**TABLE 2:** Association between LGBTQ+ status and unmet mental health needs, as well as age and race/ethnicity disparities, from 21 July 2021 to 9 May 2022.

	Model 1	Model 2	Model 3	Model 4	Model 5
LGBTQ+ (=Yes)	2.94 (2.75–3.16)***	2.32 (2.20–2.44)***	2.27 (2.18–2.39)***	2.41 (1.86–3.13)***	2.41 (2.32–2.53)***
**Socio-demographics**					
**Age**					
18–25	–	3.32 (2.97–3.67)***	4.22 (3.78–4.71)***	3.82 (3.35–4.31)***	4.18 (3.74–4.71)***
26–49	–	3.71 (3.46–3.97)***	4.26 (3.94–4.57)***	4.31 (3.94–4.71)***	4.22 (3.94–4.57)***
50–64	–	2.34 (2.18–2.48)***	2.61 (2.44–2.83)***	2.72 (2.46–2.97)***	2.61 (2.41–2.80)***
65+	–	References	References	References	References
**Race/ethnicity**					
White	–	References	References		
Black	–	0.86 (0.79–0.93)***	0.79 (0.73–0.86)***	0.79 (0.73–0.86)***	0.84 (0.76–0.91)***
Hispanic	–	0.83 (0.79–0.86)***	0.82 (0.78–0.86)***	0.83 (0.79–0.86)***	0.85 (0.79–0.92)***
Asian and others	–	0.76 (0.70–0.83)***	0.74 (0.68–0.80)***	0.74 (0.68–0.80)***	0.73 (0.67–0.78)***
**Marital status**					
Married	–	References	References	References	References
Never married	–	1.22 (1.16–1.30)***	1.31 (1.23–1.39)***	1.32 (1.25–1.40)***	1.31 (1.23–1.39)***
Widowed/divorced/separated	–	1.46 (1.40–1.52)***	1.45 (1.39–1.51)***	1.45 (1.39–1.51)***	1.45 (1.39–1.51)***
**Education attained**					
Less than high school	–	References	References	References	References
Some high school	–	0.90 (0.78–1.02).	0.84 (0.73–0.96)*	0.84 (0.72–0.96)*	0.83 (0.72–0.95)*
High school graduate or equivalent	–	1.02 (0.88–1.19)	0.93 (0.80–1.09)	0.93 (0.80–1.08)	0.93 (0.79–1.08)
Some college, but degree not received or is in progress	–	1.67 (1.42–1.95)***	1.49 (1.27–1.75)***	1.49 (1.27–1.75)***	1.49 (1.26–1.75)***
Associate’s degree	–	1.58 (1.31–1.92)***	1.42 (1.17–1.70)***	1.42 (1.17–1.70)***	1.40 (1.16–1.70)***
Bachelor’s degree	–	1.63 (1.39–1.90)***	1.57 (1.35–1.82)***	1.57 (1.35–1.82)***	1.55 (1.34–1.80)***
Graduate degree	–	1.72 (1.43–2.05)***	1.67 (1.39–1.99)***	1.67 (1.39–1.99)***	1.65 (1.38–1.97)***
**Affordability**					
**Total household income before taxes**					
0–$34,999	–	References	References	References	References
35,000–$49,999	–	0.83 (0.79–0.87)***	0.90 (0.85–0.95)***	0.90 (0.84–0.95)***	0.90 (0.84–0.95)***
50,000–$74,999	–	0.76 (0.72–0.81)***	0.90 (0.84–0.94)***	0.90 (0.84–0.94)***	0.90 (0.84–0.94)***
75,000–$99,999	–	0.68 (0.65–0.72)***	0.86 (0.83–0.90)***	0.86 (0.82–0.90)***	0.86 (0.83–0.90)***
100,000+	–	0.49 (0.46–0.53)***	0.70 (0.66–0.73)***	0.70 (0.66–0.73)***	0.70 (0.66–0.74)***
Difficulty with expenses (=Yes)	–	–	2.59 (2.51–2.66)***	2.59 (2.51–2.66)***	2.59 (2.51–2.66)***
Has public health insurance (=Yes)	–	–	1.77 (1.70–1.84)***	1.77 (1.70–1.86)***	1.77 (1.70–1.84)***
Has private health insurance (=Yes)	–	–	0.86 (0.81–0.90)***	0.85 (0.81–0.90)***	0.85 (0.81–0.90)***
**Interactions**					
LGBTQ+ (=Yes) x age (=65+)	–	–	–	References	–
LGBTQ+ (=Yes) x age (=18–25)	–	–	–	1.34 (1.03–1.75)*	–
LGBTQ+ (=Yes) x age (=26–49)	–	–	–	1.02 (0.83–1.26)	–
LGBTQ+ (= Yes) x age (= 50–64)	–	–	–	0.84 (0.66–1.06)	–
LGBTQ+ (= Yes) x race/ethnicity (=White)	–	–	–	–	References
LGBTQ+ (=Yes) x race/ethnicity (=Black)	–	–	–	–	0.72 (0.63–0.82)***
LGBTQ+ (=Yes) x race/ethnicity (=Hispanic)	–	–	–	–	0.85 (0.75–0.97)*
LGBTQ+ (=Yes) x race/ethnicity (=Asian and others)	–	–	–	–	1.07 (0.97–1.17)

Data were presented as unadjusted or adjusted odds ratios (95% confidence intervals), which were extracted from weighted logistic regression. “–” Means the corresponding covariate was not included in the regression. Besides the listed covariates, models 3, 4, and 5 also controlled state of residence. *p < 0.05, **p < 0.01, and ***p < 0.001.

Compared with LGBTQ+ who aged 65+, LGBTQ+ aged 18–25 had 1.34 times likelihood to report UMHN (AOR = 1.34, 95% CI 1.03–1.75), while no significant difference was identified in LGBTQ+ aged 26–49 (AOR = 1.02, 95% CI 0.83–1.26) and in LGBTQ+ aged 50–64 (AOR = 0.84, 95% CI 0.66–1.06) (Table 2, model 4). This higher risk of those aged 18–25 was specifically identified among lesbians (AOR = 2.29, 95% CI 1.46–3.56), but not in gay, bisexual, transgender, and queer+ (Supplementary Table 3).

Compared with White LGBTQ+ populations, Black and Hispanic LGBTQ+ had a lower likelihood to report UMHN (AOR = 0.72, 95% CI 0.63–0.82; AOR = 0.85, 95% CI 0.75–0.97, respectively), while no significant association was identified in Asian and other ethnic LGBTQ+ (AOR = 1.07, 95% CI 0.97–1.17) (Table 2, model 5). Similar associations were identified among lesbians, bisexuals, and queer+, but not among gay and transgender.

**FIGURE 1:**
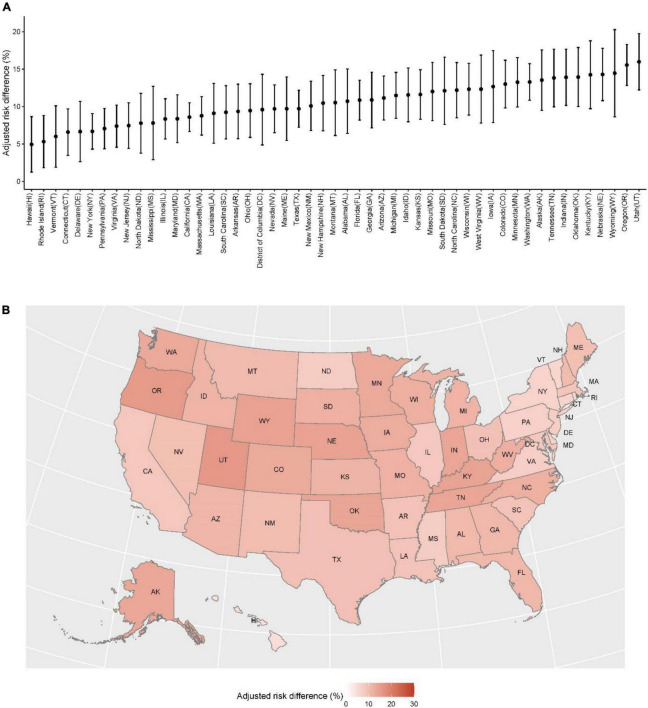
Geographic variation in the difference in unmet mental health needs between LGBTQ+ and non-LGBTQ+ (as reference) by states from 21 July 2021 to 9 May 2022. **(A)** The point presents the adjusted risk differences and the vertical line presents the 95% confidence interval, both of which were extracted from multivariable logistic regression models with LGBTQ+ status (yes vs. no) as the key predictor, controlling for socio-demographics (age, race/ethnicity, marital status, and educational attainment), and affordability (household income, difficulty with expenses, availability of public health insurance, and availability of private health insurance). **(B)** Color presents the values of adjusted risk differences, the same as those in panel **(A)**.

**FIGURE 2:**
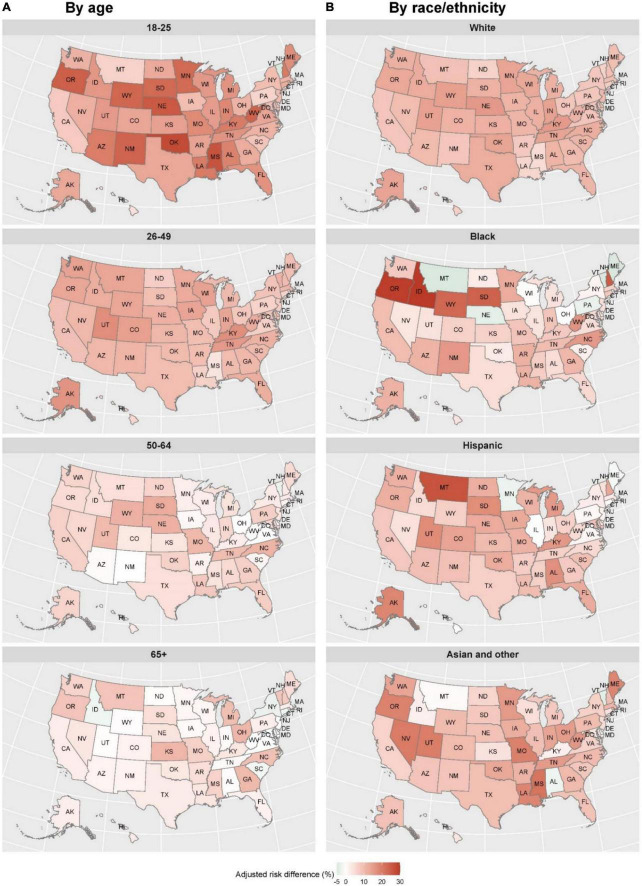
Geographic variation in the difference in unmet mental health needs between LGBTQ+ and non-LGBTQ+ (as reference) by states, age, and race/ethnicity, from 21 July 2021 to 9 May 2022. Color presents the values of adjusted risk differences. In **(A)**, the results were extracted from multivariable logistic regression models for each age group, with LGBTQ+ status (yes vs. no) as the key predictor, controlling for other covariates. In **(B)**, the results were extracted from multivariable logistic regression models for each race/ethnicity, with LGBTQ+ status (yes vs. no) as the key predictor, controlling for other covariates.

The difference in UMHN between LGBTQ+ and non-LGBTQ+ (as reference) varied substantially across states, ranging from 4.9% (95% CI 1.2–8.7%) in Hawaii to 16.0% (12.2–19.7%) in Utah (Figure 1). This substantial geographic variation primarily existed in LGBTQ+ aged 18–25 and non-White LGBTQ+ (Figure 2). The above geographic disparity was also identified in subtypes of LGBTQ+ but more obvious in transgender (Supplementary Figures 1–5).

## Discussion

### Statement of principal findings

Based on a national representative data, we identified a substantial additional risk of UMHN (2.27-time) among LGBTQ+ than non-LGBTQ+ during COVID-19 in the United States. This additional risk of UMHN was consistent in the subtypes of LGBTQ+, but highest in transgender. We also found that LGBTQ+ aged 18–25 (specifically among lesbians) was more vulnerable in terms of UMHN, while the race/ethnicity disparity was relatively small. There were wide variations across states in the risk of UMHN among LGBTQ+ as compared to non-LGBTQ+, especially among transgender individuals, LGBTQ+ aged 18–25, and non-White LGBTQ+.

### Possible explanations and comparison with other studies

The identified significant additional risk of UMHN among LGBTQ+ in comparison with non-LGBTQ+ is to some extent consistent with what the LGBTQ+ community encountered during COVID-19. During the COVID-19 crisis in the United States, LGBTQ+ populations experienced higher rates of job loss, wage reduction, and food insecurity than general populations (2, 27). These experiences of reduced resources and economic instability can be contributing factors to the disparities in UMHN identified in the study. Our supplement analysis (Supplementary Table 4) to some extent supports this point and indicates that those LGBTQ+ who have difficulty with expenses had a 1.1-time higher likelihood of reporting UMHN (AOR = 1.11, 95% CI 1.03–1.20). Our Supplementary Table 4 also highlights that the impact of job loss and wage reduction was stronger among LGBTQ+ who were relatively rich, as compared to LGBTQ+ with total household income $0–$34999, LGBTQ+ with total household income $50,000–$74,999 or $100,000+ had a higher likelihood of reporting UMHN (AOR = 1.16, 95% CI 1.04–1.28; AOR = 1.19, 95% CI 1.07–1.32, respectively). The long-lasting societal stigmatization, institutional discrimination, lack of identity-affirming mental health services, and negative personal experiences with mental health services could also contribute to the additional risk of UMHN among LGBTQ+ we found (3, 4, 11, 28–30). The identified additional risk of UMHN among LGBTQ+ during the COVID-19 crisis also keeps in line with the evidence in medical care overall. A survey from the United States also reported that a higher proportion of LGBTQ+ Americans reported difficulties accessing medical care and missing regular medical appointments than general populations (27). Our Supplementary Table 4 indicates that those LGBTQ + who had private insurance had more likelihood of reporting UMHN (AOR = 1.11, 95% CI 1.04–1.17), which may imply an unjustified attitude toward LGBTQ+ from private healthcare providers during COVID-19. To be noted, the identified gap in UMHN between LGBTQ+ and non-LGBTQ+ could be wider if considering the fact that the prevalence of mental health problems is usually higher in LGBTQ+ populations, especially the disproportionate influence on the LGBTQ+ community from COVID-19 crisis (3, 5, 6).

We also found that transgender had the highest risk of UMHN than other subtypes of LGBTQ+. This finding, to some extent, keeps in line with a study conducted in Canada, which found that after adjusting for socioeconomic variables and age, compared to cisgender heterosexual people, only the transgender but no other types of LGBTQ+ had a significant additional risk of UMHN (7). Our findings on transgender also corroborate previous studies, which concluded that compared to other subtypes of LGBTQ+, transgender people faced additional minority stressors because of their potentially visible gender expression (28, 31, 32). Our supplement analysis (Supplementary Table 4) confirms this point to some extent and indicated that those transgender people who never married had a 1.28-time likelihood of reporting UMHN (AOR = 1.28, 95% CI 1.11–1.49). During the COVID-19 crisis, the gender-affirming resources (like hormone therapy) needed by many transgender people were postponed or inaccessible, which could worsen the mental outcomes among transgender (2, 5, 33). In addition, mental health practitioners may especially express negative reactions to and reject transgender patients due to a lack of adequate training and skills toward transgender issues (30).

We further explored the UMHN among LGBTQ+ with intersecting marginalized identities and found that LGBTQ+ aged 18–25 was more vulnerable in UMHN. This could be due to that young people have relatively limited resources to enable them to overcome the barriers which adults also encounter (3, 34); the shutdown of schools or universities closed the possible gateway for young LGBTQ+ to find the mental health services they needed (11); stay-at-home and shift-to-online counseling could magnify young LGBTQ+’s concerns of “don’t want parents to know” (34, 35). Our supplement analysis (Supplementary Table 5) highlights that these limited resources owned by LGBTQ+ aged 18–25 themselves or concerns of “don’t want parents to know” may be worse among families with high household income, as Supplementary Table 5 indicates that among the group of people aged 18–25, compared to LGBTQ+ with a total household income $0–$34,999, LGBTQ+ with total household income $100,000+ had a 1.35-time likelihood of reporting UMHN (AOR = 1.35, 95% CI 1.07–1.72). However, the additional risk of UMHN among those aged 18–25 was only identified among lesbians, but not other types of LGBTQ+. No study has provided a possible explanation for this finding, as well as our supplement analysis in Supplementary Table 5. It needs more focus in future studies.

We also further explored the UMHN among LGBTQ+ with intersecting marginalized identities with a focus on people of color and found that there was a significantly lower risk of UMHN among LGBTQ+ people of color than white LGBTQ+. This finding is to some extent not consistent with a survey conducted in the United States in 2015, which found that transgender of color was less likely to have access to gender-affirming mental healthcare than White transgender (32); and a survey conducted in the United States during COVID-19, which demonstrated that the COVID-19 crisis disproportionally worsened the economic conditions on LGBTQ+ communities of color (19). The possible reason is that different from the overall results in Supplementary Table 4 and subgroup analysis of ages 18–25 (Supplementary Table 5), a higher degree of education and higher household income among people of color were associated with a lower risk of UMHN (Supplementary Tables 6, 7). The possible explanation could also be that the disproportionate higher deaths occurred in the LGBTQ+ people of color prevented them from being sampled by the survey (19), and more corresponding studies are needed.

The difference in UMHN between LGBTQ+ and non-LGBTQ+ had a wide variation across states. This geographic variation may to some extent be because of unevenly distributed LGBTQ-specific mental health services (24, 28) and different COVID-19 containment policies (like social distance rules and vaccination mandates) adopted by states (36, 37). Our state-level analysis indicated that for bisexual, states have a higher proportion of facility providing mental health treatment in a partial hospitalization/day treatment setting, offering dedicated mental health treatment program for persons with HIV or AIDS, offering vocational rehabilitation services, offering nicotine replacement therapy, offering non-nicotine smoking/tobacco cessation medications, or offering antipsychotics for the treatment of serious mental illness (SMI), was associated with a lower risk difference in reporting UMHN between bisexual and non-LGBTQ+ (Supplementary Table 8); for gay, states have a higher proportion of facility providing administrative or operational services for mental health treatment, offering vocational rehabilitation services, offering dedicated mental health treatment program for persons aged 18 years and older with SMI, or offering illness management and recovery (IMR) services, was associated with a lower risk difference in reporting UMHN between gay and non-LGBTQ+ (Supplementary Table 8); for queer, states have higher proportion of facility providing group therapy, offering mobile/off-site psychiatric crisis services, or offering antipsychotics for the treatment of SMI, was associated with a lower risk difference in reporting UMHN between queer and non-LGBTQ+ (Supplementary Table 8). We further found that the identified substantial geographic variation primarily existed among transgender individuals, LGBTQ+ aged 18–25, and LGBTQ+ people of color. Our state-level analysis indicated that among people aged 18–25, states have a higher proportion of facility providing vocational rehabilitation services, which was associated with a lower risk difference in reporting UMHN between LGBTQ+ and non-LGBTQ+ (Supplementary Table 9); among Hispanic people, states have a higher proportion of facility accepting young adults (aged 18–25 years old) for treatment, offering offers dedicated mental health treatment program for persons with HIV or AIDS, or offering dedicated mental health treatment program for LGBTQ+, was associated with lower risk difference on reporting UMHN between LGBTQ+ and non-LGBTQ+ (Supplementary Table 11). These findings highlight the necessity to put target-specific and state-specific attention or intervention on these groups.

### Strengths and limitations

To our knowledge, this is the first study to quantitatively assess the UMHN among LGBTQ+ during the COVID-19 using a national representative data. A strength of this study is the sampling of large number of participants without using sexual behavior, sexual orientation, and gender identity as sampling strategy, which could reduce the potential systematic response bias compared with research utilizing more targeted sampling methods. Moreover, the large number of participants enabled us to disaggregate LGBTQ+ participants, allowing us to provide more nuanced and practical evidence of this population. Our study is also strengthened by the inclusion of participants with a broad spectrum of intersected marginalized identities (young LGBTQ+ and LGBTQ+ people of color), thus extending our findings beyond the limited scope of most studies on LGBTQ+. Finally, our study is strengthened by the state-specific analysis, allowing for a state-specific customization of potential interventions.

The primary limitation of this study is the absence of pre-COVID data, which precluded any causal inference on how much of the identified UMHN is a result of the COVID-19 pandemic. However, this limitation can be weakened by comparing with the evidence from pre-COVID. For instance, compared to non-LGBTQ+ people, the identified risk of UMHN among LGBTQ+ people in our study is 2.27 times (AOR = 2.27, 95% CI 2.18–2.39), higher than that (AOR = 1.57; 95% CI 1.20–1.93, which was meta from ORs for transgender, bisexual, and LGQ reported in a cross-sectional based on 704 individuals in Canada in 2017 (7); compared to non-LGBTQ+ people, the identified risk of UMHN among transgender in our study is 3.63 (AOR = 3.63, 95% CI 2.97–4.39), higher than that (AOR = 2.1, 95% CI 1.3–3.3) reported in a cross-sectional study based on 704 individuals in Canada in 2017 (7).

Second, as our data are not a specific survey data on LGBTQ+, possible confounders cannot be fully controlled. For instance, the experience of stigma and discrimination, availability of identity-affirming mental health services, and availability of social support, all of which have documented impacts on access to mental health services, were not controlled and explored in this study (38–40). In addition, the HPS has no data on the reasons for UMHN; therefore, our results can only be interpreted as associations instead of causal inferences. Both limitations disable us to give strong recommendations on targeted interventions.

Third, our data are lack of clinical confirmation as data were drawn from a large-scale population survey using self-administered instruments. The existence of self-assessed-as-unnecessary will lead to some participants being grouped into no UMHN and then an inaccurate estimation of the gap in UMHN (41). In addition, lack of clinical confirmation also disabled us from distinguishing the types of needed professional mental health services (like professional mental health services from GP or specialists).

Fourth, counseling or therapy from a mental health professional is an important aspect, yet not the entirety of mental health use. Utilization of other types of mental health services, such as medication, social prescription, and other complementary and alternative treatments, could also lead to an inaccurate estimation of the gap in UMHN.

Fifth, the above three limitations disable us to put specific attention to actual needs of mental health and further disable us to explore the reasons for the gap between perceived UMHN and actual UMHN. Data collection on actual UMHN is needed in future.

Sixth, the study primarily used an Internet-based sampling strategy, which may have led to the underrepresentation of those with stronger UMHN, for example, those without permanent accommodation and people of color. A survey conducted in early of 2021 indicated that Black and Hispanic adults in the United States remain less likely than White adults to own a computer at home (42).

### Generalizability, implications, and conclusion

This study measured the additional risk of UMHN among LGBTQ+ populations than non-LGBTQ+. Subgroup evidence by age, race/ethnicity, the subtype of LGBTQ+, and states were also provided. Our evidence highlights that tailored services are needed to address specific mental vulnerabilities of different subgroups of LGBTQ+, instead of conflating sex orientation and gender identity. In addition, taking LGBTQ+ socio-demographic characteristics into consideration to reduce mental health service disparities is also necessary. Mental healthcare systems could use our evidence to ensure accessibility to professional services among LGBTQ+ populations, by customizing target-specific and state-specific interventions.

## Data availability statement

Publicly available datasets were analyzed in this study. This data can be found here: https://www.census.gov/data/experimental-data-products/household-pulse-survey.html.

## Ethics statement

The studies involving human participants were reviewed and approved by the Office of Management and Budget (approval number: 0607-1013). The patients/participants provided their written informed consent to participate in this study.

## Author contributions

SC: full access to all of the data in the study, responsibility for the integrity of the data and the accuracy of the data analysis, concept, and design. SC, YW, RS, PQ, and W-KM: acquisition, analysis, or interpretation of data. SC and YW: drafting of the manuscript, statistical analysis, administrative, technical, or material support, and supervision. SC, YW, RS, PQ, and W-KM: critical revision of the manuscript for important intellectual content. All authors contributed to the article and approved the submitted version.
