# Supplementary material for: Disparities in the unmet mental health needs between LGBTQ+ and non-LGBTQ+ populations during COVID-19 in the United States from 21 July 2021 to 9 May 2022
Source: Front Med (Lausanne). 2022 Nov 8;9:995466. doi: 10.3389/fmed.2022.995466 (PMC9679212; doi:10.3389/fmed.2022.995466)
Supplement: Supplementary file 1 [file Data_Sheet_1.docx]

**Sub Table 1. State-level measures extracted from the National Mental Health Services Survey (N-MHSS)**

| **State-level variables** | **Variable explanation** | **Response** |
| --- | --- | --- |
| a1_1_MHINTAKE | Facility offers mental health intake | yes or no |
| a1_2_MHDIAGEVAL | Facility offers mental health diagnostic evaluation | yes or no |
| a1_3_MHREFERRAL | Facility offers mental health information and/or referral | yes or no |
| a1_5_SMISEDSUD | Offered co-occurring SMI/SED and SUD | yes or no |
| a1_6_TREATMT | Facility offers substance abuse treatment | yes or no |
| a1_7_ADMINSERV | Facility offers administrative or operational services for mental health treatment | yes or no |
| a3_1_SETTINGIP | Provides mental health treatment in a 24-hour hospital inpatient setting | yes or no |
| a3_2_SETTINGRC | Provides mental health treatment in a 24-hour residential setting | yes or no |
| a3_3_SETTINGDTPH | Provides mental health treatment in a partial hospitalization/day treatment setting | yes or no |
| a3_4_SETTINGOP | Provides mental health treatment in an outpatient setting | yes or no |
| a4_1_FACILITYTYPE | Psychiatric hospital | yes or no |
| a4_2_FACILITYTYPE | Separate inpatient psychiatric unit of a general hospital | yes or no |
| a4_3_FACILITYTYPE | Residential treatment center for children | yes or no |
| a4_4_FACILITYTYPE | Residential treatment center for adults | yes or no |
| a4_5_FACILITYTYPE | Other type of residential treatment facility | yes or no |
| a4_6_FACILITYTYPE | Veterans Affairs Medical Center (VAMC) or other VA health care facility | yes or no |
| a4_7_FACILITYTYPE | Community Mental Health Center (CMHC) | yes or no |
| a4_8_FACILITYTYPE | Certified Community Behavioral Health Clinic (CCBHC) | yes or no |
| a4_9_FACILITYTYPE | Partial hospitalization/day treatment facility | yes or no |
| a4_10_FACILITYTYPE | Outpatient mental health facility | yes or no |
| a4_11_FACILITYTYPE | Multi-setting mental health facility | yes or no |
| a10_1_OWNERSHP | private for-profit organization | yes or no |
| a10_2_OWNERSHP | private non-profit organization | yes or no |
| a10_3_OWNERSHP | public agency or department | yes or no |
| a12_1_TREATPSYCHOTHRPY | Facility offers individual psychotherapy | yes or no |
| a12_2_TREATFAMTHRPY | Facility offers couples/family therapy | yes or no |
| a12_3_TREATGRPTHRPY | Facility offers group therapy | yes or no |
| a12_4_TREATCOGTHRPY | Facility offers cognitive behavioral therapy | yes or no |
| a12_5_TREATDIALTHRPY | Facility offers dialectical behavior therapy | yes or no |
| a12_6_TREATCOGREM | Facility provides cognitive remediation therapy | yes or no |
| a12_7_TREATBEHAVMOD | Facility offers behavior modification | yes or no |
| a12_8_TREATDUALMHSA | Facility offers integrated dual disorders treatment | yes or no |
| a12_9_TREATTRAUMATHRPY | Facility offers trauma therapy | yes or no |
| a12_10_TREATACTVTYTHRPY | Facility offers activity therapy | yes or no |
| a12_11_TREATELECTRO | Facility offers electroconvulsive therapy | yes or no |
| a12_12_TREATTMS | Facility provides Transcranial Magnetic Stimulation (TMS) | yes or no |
| a12_13_TREATKIT | Facility provides Ketamine Infusion Therapy (KIT) | yes or no |
| a12_14_TREATEMDR | Facility provides Eye Movement Desensitization and Reprocessing (EMDR) | yes or no |
| a12_15_TREATTELEMEDINCE | Facility offers telemedicine/telehealth therapy | yes or no |
| a13_ANTIPSYCH | Antipsychotics for the treatment of serious mental illness (SMI) | yes or no |
| a14_1_ASSERTCOMM | Facility offers assertive community treatment (ACT) | yes or no |
| a14_2_MHINTCASEMGMT | Facility offers intensive case management services | yes or no |
| a14_3_MHCASEMGMT | Facility offers case management services | yes or no |
| a14_4_MHCOURTORDERED | Facility offers court-ordered outpatient treatment | yes or no |
| a14_5_MHAOT | Facility provides assisted outpatient treatment (AOT) | yes or no |
| a14_6_MHCHRONIC | Facility offers chronic disease/illness management services | yes or no |
| a14_7_ILLNESSMGMT | Facility offers illness management and recovery (IMR) services | yes or no |
| a14_8_PRIMARYCARE | Facility offers integrated primary care services | yes or no |
| a14_9_DIETEXERCOUNSEL | Facility offers diet and exercise counseling | yes or no |
| a14_10_FAMPSYCHED | Facility offers family psychoeducation | yes or no |
| a14_11_MHEDUCATION | Facility offers education services | yes or no |
| a14_12_MHHOUSING | Facility offers housing services | yes or no |
| a14_13_SUPPHOUSING | Facility offers supported housing programs | yes or no |
| a14_14_MHPSYCHREHAB | Facility offers psychosocial rehabilitation services | yes or no |
| a14_15_MHVOCREHAB | Facility offers vocational rehabilitation services | yes or no |
| a14_16_SUPPEMPLOY | Facility offers supported employment services | yes or no |
| a14_17_FOSTERCARE | Facility offers therapeutic foster care | yes or no |
| a14_18_MHLEGAL | Facility offers legal advocacy | yes or no |
| a14_19_MHEMGCY | Facility offers psychiatric emergency walk-in services | yes or no |
| a14_20_MHSUICIDE | Facility offers suicide prevention services | yes or no |
| a14_21_MHCONSUMER | Facility offers consumer-run (peer support) services | yes or no |
| a14_22_MHHBV | Facility provides testing for Hepatitis B (HBV) | yes or no |
| a14_23_MHHCV | Facility provides testing for Hepatitis C (HCV) | yes or no |
| a14_24_MHHIV | Facility provides HIV testing | yes or no |
| a14_25_MHSTD | Facility provides STD testing | yes or no |
| a14_26_MHTB | Facility provides TB screening | yes or no |
| a14_27_MHTOBACCOUSE | Facility offers screening for tobacco use | yes or no |
| a14_28_MHTOBACCOCESS | Facility offers smoking/tobacco cessation counseling | yes or no |
| a14_29_MHNICOTINEREP | Facility offers nicotine replacement therapy | yes or no |
| a14_30_SMOKINGCESSATION | Facility offers non-nicotine smoking/tobacco cessation medications | yes or no |
| a17_1_YNGCHLD | Accepts young children (aged 0-5 years old) for treatment | yes or no |
| a17_2_CHILDREN | Accepts children (aged 6-12 years old) for treatment | yes or no |
| a17_3_ADOLES | Accepts adolescents (aged 13-17 years old) for treatment | yes or no |
| a17_4_YOUNGADULTS | Accepts young adults (aged 18-25 years old) for treatment | yes or no |
| a17_5_ADULT | Accepts adults (aged 26-64 years old) for treatment | yes or no |
| a17_6_SENIORS | Accepts seniors (aged 65 years and older) for treatment | yes or no |
| a18_1_SED | Facility offers dedicated mental health treatment program for children/adolescent | yes or no |
| a18_2_TAYOUNGADULTS | Facility offers dedicated mental health treatment program for young adults | yes or no |
| a18_3_SPMI | Facility offers dedicated mental health treatment program for persons aged 18 and older with serious mental illness (SMI) | yes or no |
| a18_4_SRVC63 | Facility offers dedicated mental health treatment program for seniors or older adults | yes or no |
| a18_5_ALZHDEMENTIA | Facility has a tailored program for persons with Alzheimer's or dementia | yes or no |
| a18_6_SRVC31 | Facility offers dedicated mental health treatment program for persons with co-occurring mental and substance use disorders | yes or no |
| a18_7_SPECGRPEATING | Facility offers dedicated mental health treatment program for persons with eating disorders | yes or no |
| a18_8_FIRSTEPPSYCH | Facility offers dedicated mental health treatment program for persons experiencing first-episode psychosis | yes or no |
| a18_9_SRVC122 | Facility offers dedicated mental health treatment program for persons who have experienced intimate partner violence, domestic violence | yes or no |
| a18_10_POSTTRAUM | Facility offers a dedicated mental health treatment program for persons with a diagnosis of post-traumatic stress disorder (PTSD) | yes or no |
| a18_11_SRVC116 | Facility offers dedicated mental health treatment program for persons who have experienced trauma (excluding persons with a PTSD diagnosis) | yes or no |
| a18_12_TRAUMATICBRAIN | Facility offers dedicated mental health treatment program for persons with traumatic brain injury (TBI) | yes or no |
| a18_13_SRVC113 | Facility offers dedicated mental health treatment program for veterans | yes or no |
| a18_14_SRVC114 | Facility offers dedicated mental health treatment program for active duty military | yes or no |
| a18_15_SRVC115 | Facility offers dedicated mental health treatment program for members of military families | yes or no |
| a18_16_SRVC62 | Facility offers dedicated mental health treatment program for LGBTQ | yes or no |
| a18_17_SRVC61 | Facility offers dedicated mental health treatment program for forensic clients | yes or no |
| a18_18_SRVC32 | Facility offers dedicated mental health treatment program for persons with HIV or AIDS | yes or no |
| a19_CRISISTEAM2 | Facility offers crisis intervention team that handles acute mental health issues | yes or no |
| a20_PSYCHON | Services for psychiatric emergencies onsite | yes or no |
| a21_PSYCHOFF | Mobile/off-site psychiatric crisis services | yes or no |
| a22_SIGNLANG | Provides mental health treatment services in sign language for the deaf and hard | yes or no |
| a23_LANG | Facility provides mental health treatment services in a language other than English | yes or no |
| a24_1_CONTED | Continuing education requirements for professional staff is part of this facility’s standard operating procedures | yes or no |
| a24_2_CASEREV | Regularly scheduled case review with a supervisor is part of this facility’s standard operating procedures | yes or no |
| a24_3_QUALREV | Regularly scheduled case review by an appointed quality review committee is part of this facility’s standard operating procedures | yes or no |
| a24_4_OUTFUP | Client outcome follow-up after discharge is part of this facility’s standard operating procedures | yes or no |
| a24_5_CQIP | Continuous quality improvement processes is part of this facility’s standard operating procedures | yes or no |
| a24_6_SATSUR | Periodic client satisfaction surveys are part of this facility’s standard operating procedures | yes or no |
| a24_7_CPPR | Clinical provider peer review (CPPR) is part of this facility’s standard operating procedures | yes or no |
| a24_8_RCA | Root cause analysis (RCA) is part of facility's standard operating procedures | yes or no |
| a26_USEDSECLUSION | Staff have used seclusion or restraint with clients in the 12-month period | yes or no |
| a26_a_ADOPTSECLUSION | Facility has policies to minimize the use of seclusion or restraint | yes or no |
| a28_FEESCALE | Facility uses a sliding fee scale | yes or no |
| a29_PAYASST | Facility offers treatment at no charge or minimal payment to clients who cannot | yes or no |
| a30_1_REVCHK1 | Accepts cash or self-payment for mental health treatment services | yes or no |
| a30_2_REVCHK2 | Accepts private health insurance as source of payment for mental health treatment | yes or no |
| a30_3_REVCHK8 | Accepts Medicare as source of payment for mental health treatment services | yes or no |
| a30_4_REVCHK5 | Accepts Medicaid as source of payment for mental health treatment services | yes or no |
| a30_5_REVCHK10 | Accepts state-financed health insurance plan other than Medicaid as source of payment | yes or no |
| a30_6_FUNDSMHA | Accepts state mental health agency funds as source of payment for mental health | yes or no |
| a30_7_FUNDSTATEWELFARE | Accepts state welfare or child and family services agency funds as source of payment | yes or no |
| a30_8_FUNDSTATEJUV | Accepts state corrections/juvenile justice agency funds as source of payment | yes or no |
| a30_9_FUNDSTATEEDUC | Accepts state education agency funds as source of payment for mental health treatment | yes or no |
| a30_10_FUNDOTHSTATE | Accepts other state government funds as source of payment for mental health treatment | yes or no |
| a30_11_FUNDLOCALGOV | Accepts county or local government funds as source of payment for mental health treatment | yes or no |
| a30_12_FUNDCSBG | Accepts Community Service Block Grants as source of payment for mental health treatment | yes or no |
| a30_13_FUNDCMHG | Accepts Community Mental Health Block Grants as source of payment formental health treatment | yes or no |
| a30_14_FUNDFEDGRANT | Accepts Federal grants as source of payment for mental health treatment services | yes or no |
| a30_15_REVCHK15 | Accepts federal military insurance (such as TRICARE) as source of payment for mental health treatment | yes or no |
| a30_16_FUNDVA | Accepts U.S. Department of Veterans Affairs funds as source of payment for mental health treatment | yes or no |
| a30_17_REVCHK17 | Accepts IHS/Tribal/Urban (ITU) funds as source of payment for mental health treatment | yes or no |
| a30_18_FUNDPRIVCOMM | Accepts Private or Community foundation funds as source of payment for mental health treatment | yes or no |
| smoke_allow | Permitted to smoke outside or within any building | yes or no |
| license | Facility have licensing, certification, or accreditation | yes or no |

**Sub Table 2. Adjusted odds ratios on unmet mental health needs in the U.S. from July 21, 2021 to May 9, 2022, by subtype of LGBTQ+**

|  | **Adjusted Odds ratio (95% CI)** |
| --- | --- |
| **Lesbian^a^** | 2.27(2.01-2.59)*** |
| **Gay^b^** | 1.75(1.51-2.03)*** |
| **Bisexual^c^** | 2.80(2.69-2.92)*** |
| **Transgender^d^** | 3.63(2.97-4.39)*** |
| **Queer+^e^** | 1.99(1.84-2.16)*** |

a, results were extracted from a multivariable logistic regression model, with lesbian or non-LGBTQ+ as the key predictor, controlling for socio-demographics (age, race/ethnicity, marital status, and educational attainment), affordability (household income, difficulty with expenses, availability of public health insurance, and availability of private health insurance), and state of residence.

b, results were extracted from a multivariable logistic regression model, with gay or non-LGBTQ+ as the key predictor, controlling for the same covariates.

c, results were extracted from a multivariable logistic regression model, with bisexual or non-LGBTQ+ as the key predictor, controlling for the same covariates.

d, results were extracted from a multivariable logistic regression model, with transgender or non-LGBTQ+ as the key predictor, controlling for the same covariates.

e, results were extracted from a multivariable logistic regression model, with queer+ or non-LGBTQ+ as the key predictor, controlling for the same covariates.

**Sub Table 3. Age and race/ethnicity disparities in** **unmet mental health needs in the U.S. from July 21, 2021 to May 9, 2022, by subtype of LGBTQ+**

|  | **Adjusted Odds ratio (95% CI)** |  | **Adjusted Odds ratio (95% CI)** |
| --- | --- | --- | --- |
| **Model 1** |  | **Model 2** |  |
| Lesbian (= Yes) x age (= 65+) | Reference | Lesbian (= Yes) x race/ethnicity (= White) | Reference |
| Lesbian (= Yes) x age (= 18-25) | **2.29(1.46-3.56)***** | Lesbian (= Yes) x race/ethnicity (= Black) | **0.67(0.49-0.92)*** |
| Lesbian (= Yes) x age (= 26-49) | 1.23(0.77-1.97) | Lesbian (= Yes) x race/ethnicity (= Hispanic) | 0.84(0.63-1.11) |
| Lesbian (= Yes) x age (= 50-64) | 1.07(0.66-1.75) | Lesbian (= Yes) x race/ethnicity (= Asian and other) | 1.30(0.89-1.88) |
| **Model 3** |  | **Model 4** |  |
| Gay (= Yes) x age (= 65+) | Reference | Gay (= Yes) x race/ethnicity (= White) | Reference |
| Gay (= Yes) x age (= 18-25) | 1.30(0.63-2.66) | Gay (= Yes) x race/ethnicity (= Black) | 1.01(0.71-1.42) |
| Gay (= Yes) x age (= 26-49) | 0.90(0.45-1.77) | Gay (= Yes) x race/ethnicity (= Hispanic) | 1.06(0.77-1.46) |
| Gay (= Yes) x age (= 50-64) | 0.83(0.43-1.60) | Gay (= Yes) x race/ethnicity (= Asian and other) | 0.82(0.63-1.08) |
| **Model 5** |  | **Model 6** |  |
| Bisexual (= Yes) x age (= 65+) | Reference | Bisexual (= Yes) x race/ethnicity (= White) | Reference |
| Bisexual (= Yes) x age (= 18-25) | 1.35(0.80-2.29) | Bisexual (= Yes) x race/ethnicity (= Black) | **0.71(0.59-0.84)***** |
| Bisexual (= Yes) x age (= 26-49) | 1.16(0.69-1.95) | Bisexual (= Yes) x race/ethnicity (= Hispanic) | 0.92(0.79-1.08) |
| Bisexual (= Yes) x age (= 50-64) | 1.06(0.60-1.88) | Bisexual (= Yes) x race/ethnicity (= Asian and other) | 1.19(0.92-1.51) |
| **Model 7** |  | **Model 8** |  |
| Transgender (= Yes) x age (= 65+) | Reference | Transgender (= Yes) x race/ethnicity (= White) | Reference |
| Transgender (= Yes) x age (= 18-25) | 0.66(0.28-1.57) | Transgender (= Yes) x race/ethnicity (= Black) | 0.55(0.25-1.22) |
| Transgender (= Yes) x age (= 26-49) | 0.61(0.30-1.26) | Transgender (= Yes) x race/ethnicity (= Hispanic) | 0.90(0.51-1.57) |
| Transgender (= Yes) x age (= 50-64) | 0.68(0.27-1.73) | Transgender (= Yes) x race/ethnicity (= Asian and other) | 1.17(0.82-1.68) |
| **Model 9** |  | **Model 10** |  |
| Queer+ (= Yes) x age (= 65+) | Reference | Queer+ (= Yes) x race/ethnicity (= White) | Reference |
| Queer+ (= Yes) x age (= 18-25) | 0.88(0.62-1.26) | Queer+ (= Yes) x race/ethnicity (= Black) | **0.70(0.52-0.92)*** |
| Queer+ (= Yes) x age (= 26-49) | 0.74(0.52-1.04). | Queer+ (= Yes) x race/ethnicity (= Hispanic) | **0.78(0.64-0.94)*** |
| Queer+ (= Yes) x age (= 50-64) | **0.66(0.49-0.88)**** | Queer+ (= Yes) x race/ethnicity (= Asian and other) | 1.02(0.84-1.26) |

In model 1, results were extracted from a multivariable logistic regression model, with interaction lesbian (yes or non-LGBTQ+) and age as the key predictor, controlling for lesbian (yes or non-LGBTQ+), socio-demographics (age, race/ethnicity, marital status, and educational attainment), affordability (household income, difficulty with expenses, availability of public health insurance, and availability of private health insurance), and state of residence.

In model 2, results were extracted from a multivariable logistic regression model, with interaction lesbian (yes or non-LGBTQ+) and race/ethnicity as the key predictor, controlling for lesbian (yes or non-LGBTQ+), race/ethnicity, and other covariates.

In model 3, results were extracted from a multivariable logistic regression model, with interaction gay (yes or non-LGBTQ+) and age as the key predictor, controlling for gay (yes or non-LGBTQ+), age, and other covariates.

In model 4, results were extracted from a multivariable logistic regression model, with interaction gay (yes or non-LGBTQ+) and race/ethnicity as the key predictor, controlling for gay (yes or non-LGBTQ+), race/ethnicity, and other covariates.

In model 5, results were extracted from a multivariable logistic regression model, with interaction bisexual (yes or non-LGBTQ+) and age as the key predictor, controlling for bisexual (yes or non-LGBTQ+), age, and other covariates.

In model 6, results were extracted from a multivariable logistic regression model, with interaction bisexual (yes or non-LGBTQ+) and race/ethnicity as the key predictor, controlling for bisexual (yes or non-LGBTQ+), race/ethnicity, and other covariates.

In model 7, results were extracted from a multivariable logistic regression model, with interaction transgender (yes or non-LGBTQ+) and age as the key predictor, controlling for transgender (yes or non-LGBTQ+), age, and other covariates.

In model 8, results were extracted from a multivariable logistic regression model, with interaction transgender (yes or non-LGBTQ+) and race/ethnicity as the key predictor, controlling for transgender (yes or non-LGBTQ+), race/ethnicity, and other covariates.

In model 9, results were extracted from a multivariable logistic regression model, with interaction queer+ (yes or non-LGBTQ+) and age as the key predictor, controlling for queer+ (yes or non-LGBTQ+), age, and other covariates.

In model 10, results were extracted from a multivariable logistic regression model, with interaction queer+ (yes or non-LGBTQ+) and race/ethnicity as the key predictor, controlling for queer+ (yes or non-LGBTQ+), race/ethnicity, and other covariates.

**Sub Figure 1. Geographic Variation in the difference of unmet mental health needs between lesbian and non-LGBTQ+ (as reference) by state, age, and race/ethnicity, from July 21, 2021 to May 9, 2022.** Colour present the values of adjusted risk differences. In panel A, results were extracted from multivariable logistic regression models for each age group, with lesbian or non-LGBTQ+ as the key predictor, controlling for other covariates. In panel B, results were extracted from multivariable logistic regression models for each race/ethnicity, with lesbian or non-LGBTQ+ as the key predictor, controlling for other covariates.


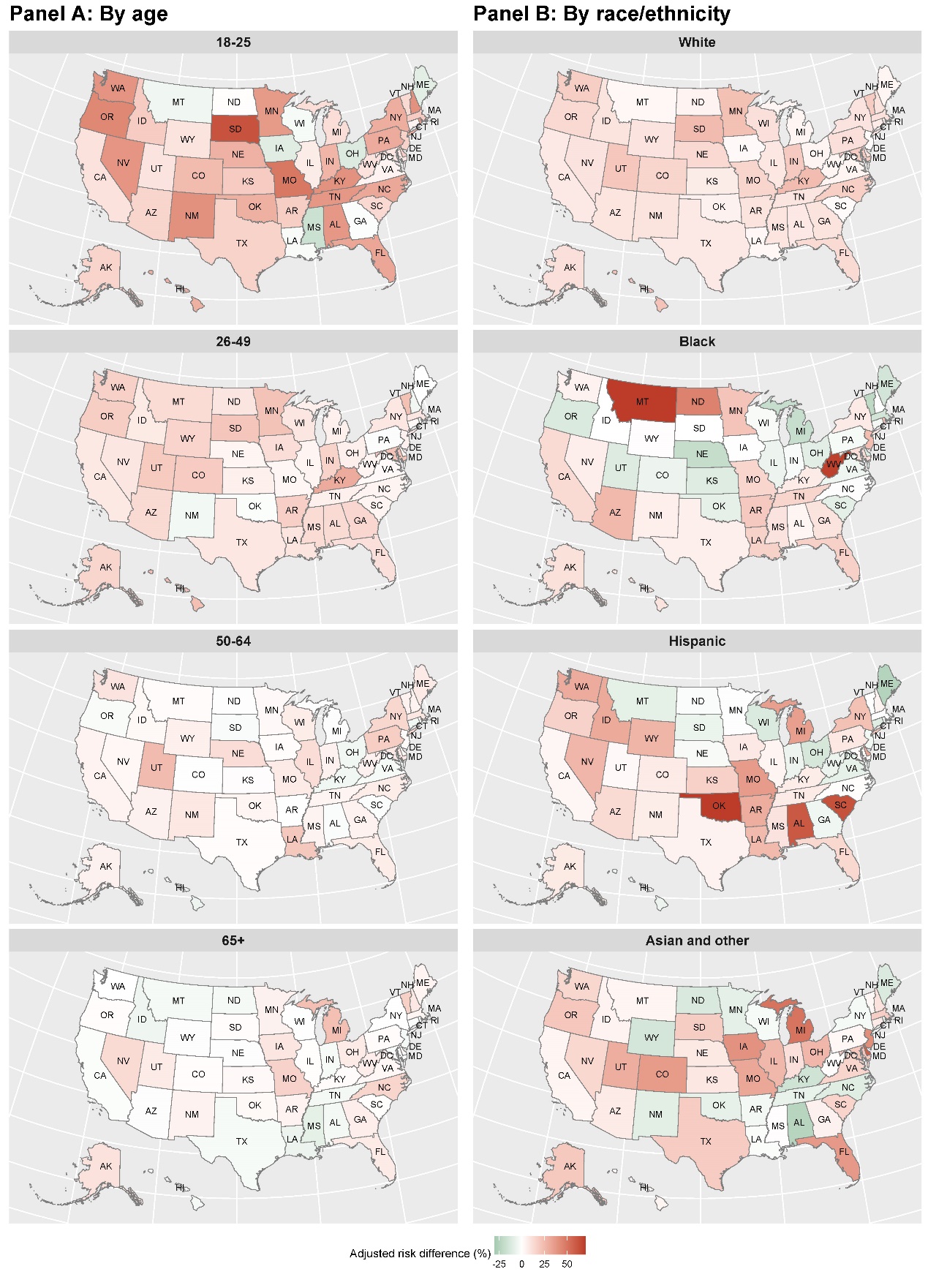


**Sub Figure 2. Geographic Variation in the difference of unmet mental health needs between gay and non-LGBTQ+ (reference) by state, age, and race/ethnicity, from July 21, 2021 to May 9, 2022.** Colour present the values of adjusted risk differences. In panel A, results were extracted from multivariable logistic regression models for each age group, with gay or non-LGBTQ+ as the key predictor, controlling for other covariates. In panel B, results were extracted from multivariable logistic regression models for each race/ethnicity, with gay or non-LGBTQ+ as the key predictor, controlling for other covariates.


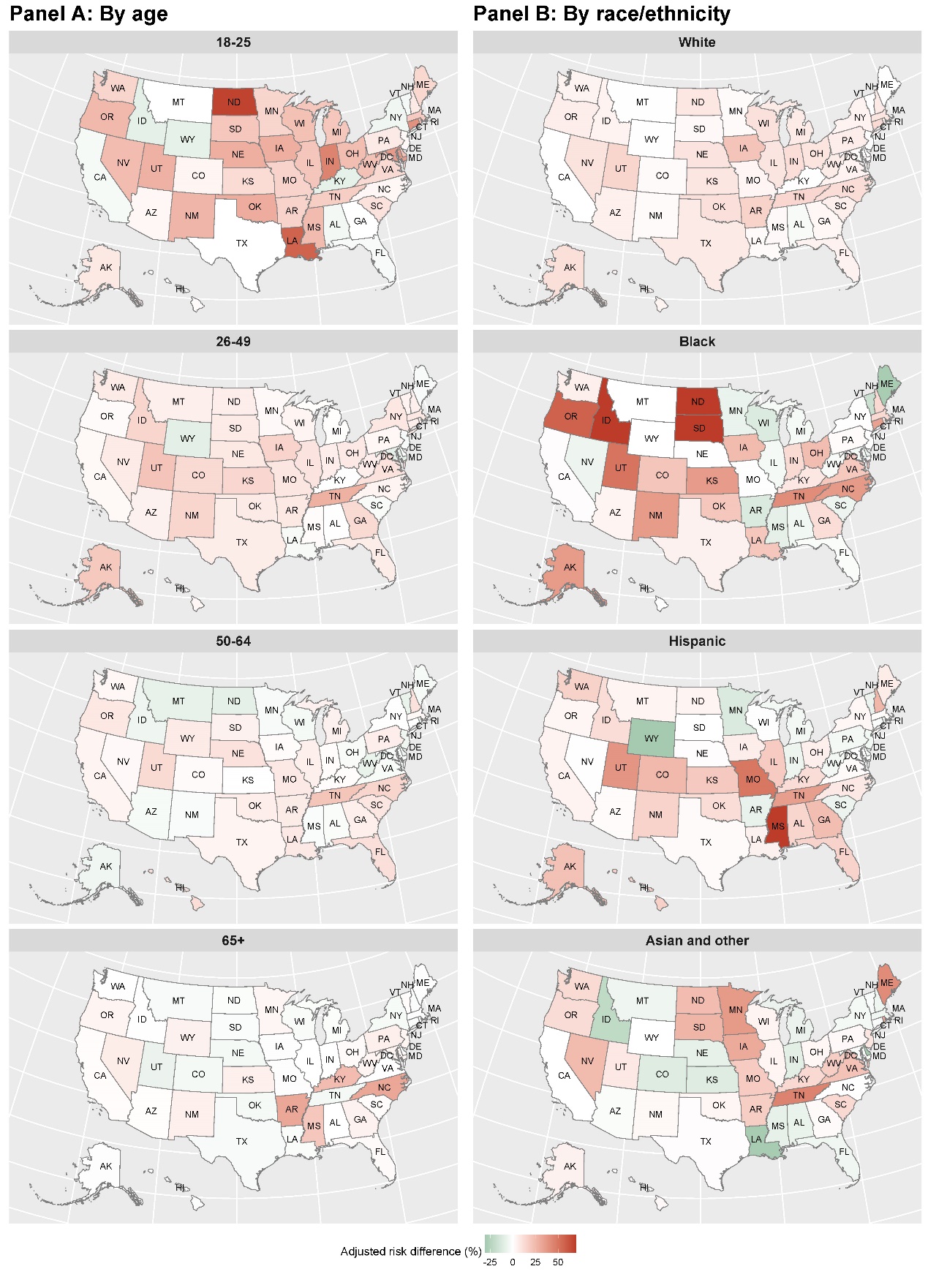


**Sub Figure 3. Geographic Variation in the gap of unmet mental health needs between bisexual and non-LGBTQ+ (reference) by state, age, and race/ethnicity, from July 21, 2021 to May 9, 2022.** Colour present the values of adjusted risk differences. In panel A, results were extracted from multivariable logistic regression models for each age group, with bisexual or non-LGBTQ+ as the key predictor, controlling for other covariates. In panel B, results were extracted from multivariable logistic regression models for each race/ethnicity, with bisexual or non-LGBTQ+ as the key predictor, controlling for other covariates.


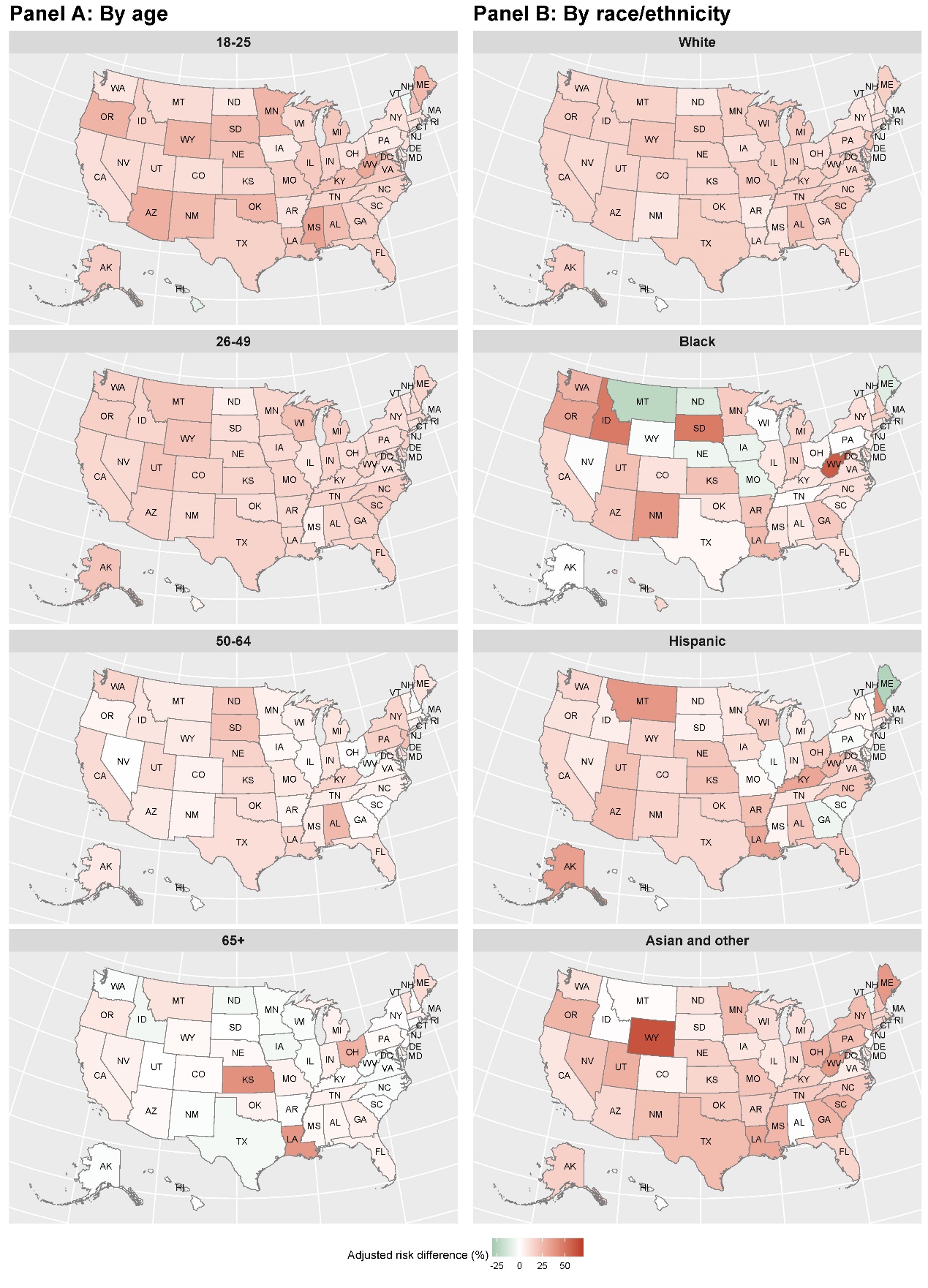


**Sub Figure 4. Geographic Variation in the gap of unmet mental health needs between transgender and non-LGBTQ+ (reference) by state, age, and race/ethnicity, from July 21, 2021 to May 9, 2022.** Colour present the values of adjusted risk differences. In panel A, results were extracted from multivariable logistic regression models for each age group, with transgender or non-LGBTQ+ as the key predictor, controlling for other covariates. In panel B, results were extracted from multivariable logistic regression models for each race/ethnicity, with transgender or non-LGBTQ+ as the key predictor, controlling for other covariates.


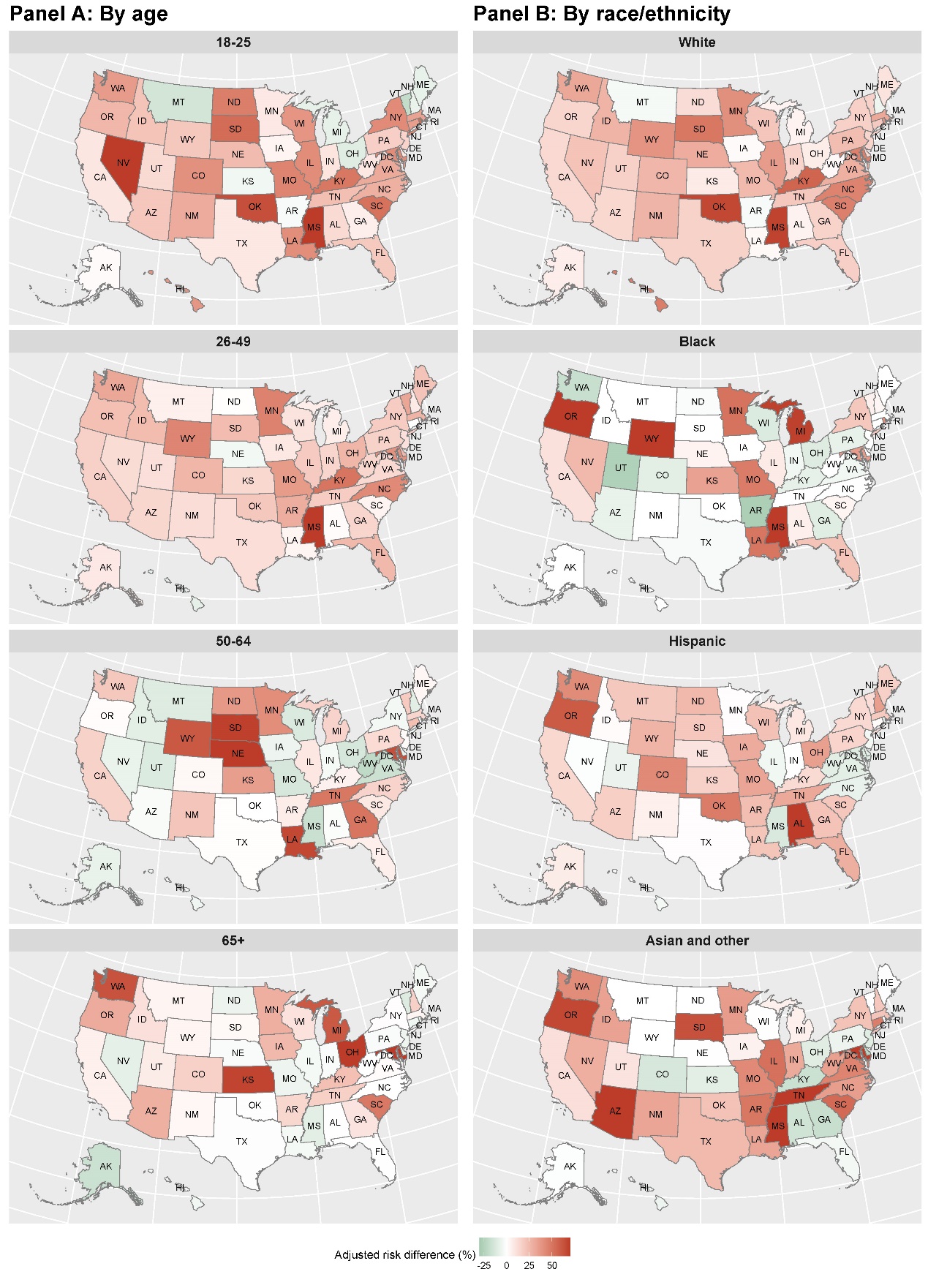


**Sub Figure 5. Geographic Variation in the gap of unmet mental health needs between queer+ and non-LGBTQ+ (reference) by state, age, and race/ethnicity, from July 21, 2021 to May 9, 2022.** Colour present the values of adjusted risk differences. In panel A, results were extracted from multivariable logistic regression models for each age group, with queer+ or non-LGBTQ+ as the key predictor, controlling for other covariates. In panel B, results were extracted from multivariable logistic regression models for each race/ethnicity, with queer+ or non-LGBTQ+ as the key predictor, controlling for other covariates.


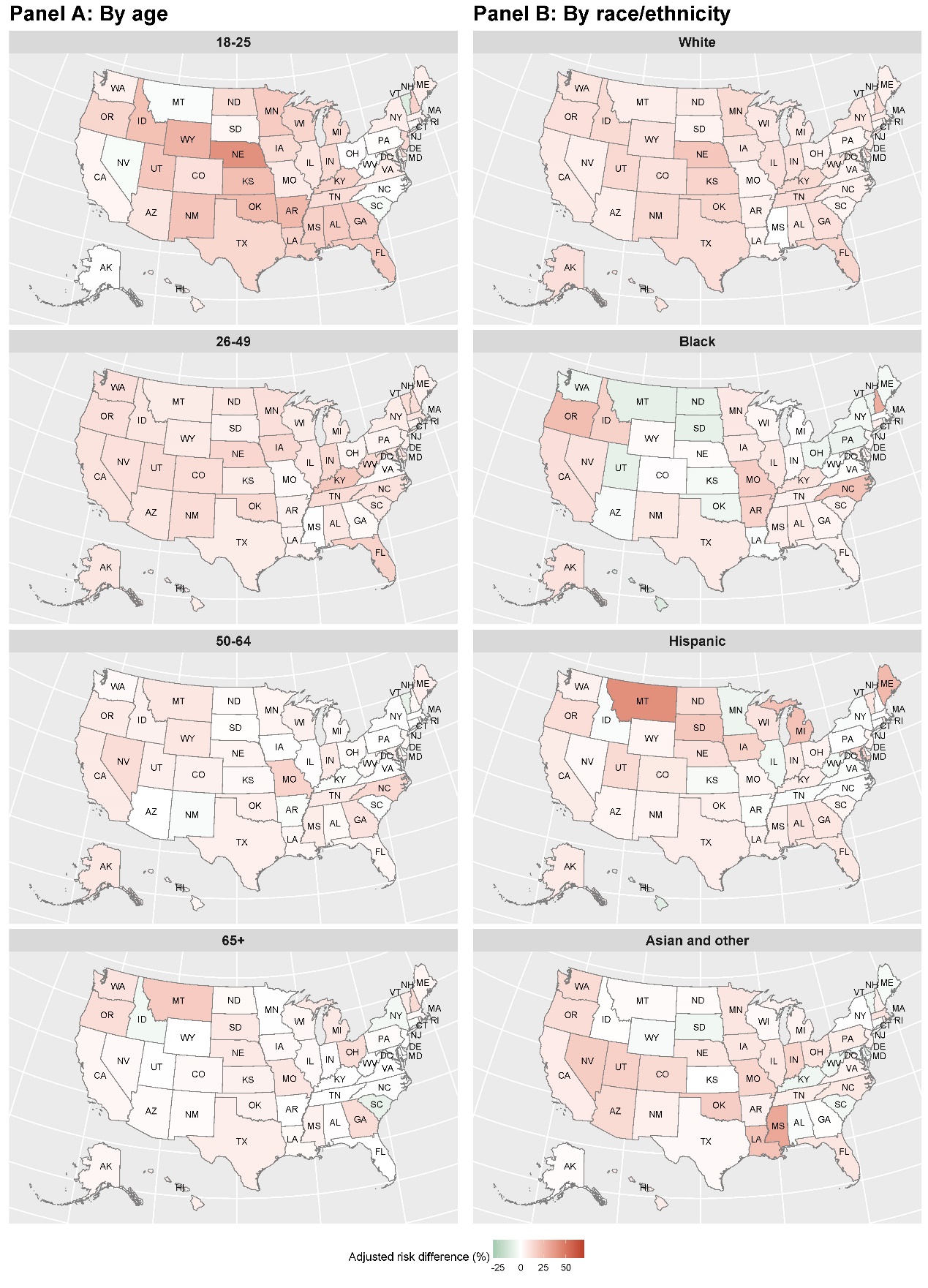


**Sub Table 4. Interaction effects of modifiable covariates on the association between LGBTQ+ status and unmet mental health needs.** Only the results for interaction were presented. Data were presented as adjusted odds ratios (95% confidence intervals), which were extracted from weighted logistic regression. In the fitted weighted logistic regression, the outcome is the unmet mental health needs (yes or no), and the independent variables include, objective (shown in columns 2-7), age, race/ethnicity, marital status and its interaction with objective, educational attainment and its interaction with objective, household income and its interaction with objective, difficulty with expenses and its interaction with objective, availability of public health insurance and its interaction with objective, availability of private health insurance and its interaction with objective, and state of residence. * p < 0.05, ** p < 0.01, *** p < 0.001

|  | **Objective** | | | | | |
| --- | --- | --- | --- | --- | --- | --- |
|  | **LGBTQ+** | **Lesbian** | **Gay** | **Bisexual** | **Transgender** | **Queer+** |
| **Marital Status** |  |  |  |  |  |  |
| Objective:Married | Reference | Reference | Reference | Reference | Reference | Reference |
| Objective:Never married | 0.97(0.89-1.05) | 1.19(0.98-1.45) | 0.79(0.61-1.02) | **0.79(0.68-0.90)**** | **1.28(1.11-1.49)**** | **0.54(0.37-0.79)**** |
| Objective:Widowed/divorced/separated | 0.90(0.80-1.01) | 0.80(0.59-1.07) | 0.95(0.68-1.34) | **0.73(0.61-0.88)**** | 1.13(0.94-1.35) | **0.44(0.25-0.79)*** |
| **Total household income before taxes** |  |  |  |  |  |  |
| Objective:$0-$34999 | Reference | Reference | Reference | Reference | Reference | Reference |
| Objective:$35,000 - $49,999 | 1.03(0.89-1.20) | 0.96(0.71-1.28) | 0.99(0.68-1.43) | 1.06(0.92-1.23) | 0.99(0.77-1.27) | 1.30(0.71-2.34) |
| Objective:$50,000 - $74,999 | **1.16(1.04-1.28)*** | 1.40(1.02-1.92). | 1.06(0.85-1.32) | 1.15(0.96-1.38) | 1.16(1.00-1.35). | 0.82(0.47-1.43) |
| Objective:$75,000 - $99,999 | 1.04(0.90-1.20) | 0.98(0.76-1.26) | 0.88(0.61-1.28) | 1.04(0.84-1.31) | 1.09(0.88-1.38) | 0.71(0.44-1.14) |
| Objective:$100000+ | **1.19(1.07-1.32)**** | 1.13(0.75-1.70) | 0.88(0.63-1.23) | **1.21(1.07-1.38)*** | **1.45(1.21-1.73)**** | 1.63(0.97-2.75). |
| **Education attained** |  |  |  |  |  |  |
| Objective:Less than high school | Reference | Reference | Reference | Reference | Reference | Reference |
| Objective:Some high school | 1.07(0.64-1.79) | 1.40(0.44-4.44) | 1.21(0.53-2.77) | 0.89(0.41-1.90) | 0.87(0.44-1.72) | 0.90(0.41-1.99) |
| Objective:High school graduate or equivalent | 1.15(0.87-1.54) | 1.09(0.38-3.13) | 1.07(0.46-2.46) | 0.93(0.50-1.75) | 1.02(0.75-1.40) | 1.16(0.58-2.36) |
| Objective:Some college, but degree not received or is in progress | 1.16(0.85-1.58) | 1.04(0.34-3.16) | 1.23(0.52-2.94) | 0.83(0.44-1.52) | 1.11(0.76-1.62) | 0.96(0.47-1.97) |
| Objective:Associate’s degree | 1.16(0.84-1.60) | 0.96(0.31-3.00) | 1.09(0.45-2.66) | 0.81(0.42-1.57) | 1.28(0.94-1.73) | 0.66(0.22-1.95) |
| Objective:Bachelor's degree | 0.96(0.76-1.23) | 0.85(0.30-2.41) | 0.75(0.33-1.70) | 0.70(0.39-1.27) | 1.05(0.81-1.36) | 0.79(0.40-1.55) |
| Objective:Graduate degree | 0.84(0.63-1.14) | 0.74(0.24-2.25) | 0.80(0.32-2.01) | 0.55(0.30-1.02). | 1.03(0.75-1.43) | 0.73(0.33-1.62) |
| **Objective:Difficulty with expenses (= Yes)** | **1.11(1.03-1.20)*** | 1.03(0.83-1.27) | 1.00(0.84-1.21) | 1.06(0.76-1.48) | **1.22(1.07-1.38)**** | 1.05(0.96-1.16) |
| **Objective:Has public health insurance (=Yes)** | 0.90(0.78-1.02) | 0.84(0.66-1.07) | 0.82(0.64-1.04) | 0.89(0.74-1.07) | 0.94(0.79-1.11) | 1.15(0.79-1.68) |
| **Objective:Has private health insurance (=Yes)** | **1.11(1.04-1.17)**** | **1.27(1.03-1.58)*** | 0.96(0.80-1.14) | 1.09(1.00-1.19). | **1.15(1.03-1.28)*** | 1.25(0.86-1.79) |

**Sub Table 5. Interaction effects of modifiable covariates on the association between LGBTQ+ status and unmet mental health needs, among those aged 18-25.** Only the results for interaction were presented. Data were presented as adjusted odds ratios (95% confidence intervals), which were extracted from weighted logistic regression. In the fitted weighted logistic regression, the outcome is the unmet mental health needs (yes or no), and the independent variables include, objective (shown in columns 2-7), race/ethnicity, marital status and its interaction with objective, educational attainment and its interaction with objective, household income and its interaction with objective, difficulty with expenses and its interaction with objective, availability of public health insurance and its interaction with objective, availability of private health insurance and its interaction with objective, and state of residence. “-“ means no corresponding result. * p < 0.05, ** p < 0.01, *** p < 0.001

|  | **Objective** | | | | | |
| --- | --- | --- | --- | --- | --- | --- |
|  | **LGBTQ+** | **Lesbian** | **Gay** | **Bisexual** | **Transgender** | **Queer+** |
| **Marital Status** |  |  |  |  |  |  |
| Objective:Married | Reference | Reference | Reference | Reference | Reference | Reference |
| Objective:Never married | 0.81(0.46-1.40) | 1.13(0.46-2.80) | 0.24(0.06-1.00). | 0.67(0.38-1.17) | 0.44(0.20-1.00). | 1.62(0.79-3.32) |
| Objective:Widowed/divorced/separated | 0.84(0.42-1.67) | 0.05(0.03-1.57) | - | 1.21(0.49-3.00) | 0.20(0.03-1.54) | 1.00(0.28-3.60) |
| **Total household income before taxes** |  |  |  |  |  |  |
| Objective:$0-$34999 | Reference | Reference | Reference | Reference | Reference | Reference |
| Objective:$35,000 - $49,999 | 1.08(0.67-1.75) | 0.93(0.39-2.27) | 0.80(0.26-2.51) | 1.21(0.66-2.18) | 1.63(0.73-3.67) | 0.98(0.53-1.80) |
| Objective:$50,000 - $74,999 | 1.03(0.79-1.35) | 2.29(0.83-6.36) | 1.48(0.66-3.29) | 1.04(0.64-1.72) | 0.83(0.38-1.77) | 0.70(0.39-1.23) |
| Objective:$75,000 - $99,999 | 0.85(0.61-1.17) | 0.76(0.38-1.55) | 0.75(0.30-1.88) | 0.94(0.59-1.49) | 0.44(0.19-1.03). | 0.73(0.41-1.31) |
| Objective:$100000+ | **1.35(1.07-1.72)*** | 1.62(0.77-3.39) | 1.88(0.92-3.82) | 1.36(0.96-1.93). | 1.95(1.03-3.74). | 1.06(0.63-1.79) |
| **Education attained** |  |  |  |  |  |  |
| Objective:Less than high school | Reference | Reference | Reference | Reference | Reference | Reference |
| Objective:Some high school | 3.25(0.58-18.54) | 4.71(0.30-72.97) | 1.39(0.13-14.73) | 1.45(0.20-10.70) | 5.47(0.42-72.24) | 3.67(0.59-22.65) |
| Objective:High school graduate or equivalent | **3.16(1.17-8.58)*** | 4.14(0.30-56.26) | 1.08(0.10-11.70) | 1.46(0.40-5.37) | 4.90(0.63-38.86) | **4.81(1.36-16.78)*** |
| Objective:Some college, but degree not received or is in progress | 2.56(0.84-7.77) | 1.73(0.15-20.49) | 1.84(0.21-16.44) | 1.14(0.28-4.71) | 3.94(0.35-43.82) | 4.26(1.07-16.95). |
| Objective:Associate’s degree | 2.25(0.67-7.54) | 1.72(0.14-21.54) | 1.40(0.12-17.29) | 0.85(0.20-3.74) | 1.55(0.07-34.12) | **5.47(1.23-24.53)*** |
| Objective:Bachelor's degree | 1.86(0.64-5.47) | 1.55(0.14-16.78) | 0.49(0.06-4.14) | 0.94(0.22-3.97) | 2.77(0.26-29.37) | 2.72(0.75-9.87) |
| Objective:Graduate degree | 3.03(1.04-8.76). | 1.22(0.09-17.12) | 0.52(0.04-6.05) | 1.60(0.40-6.36) | 3.00(0.29-31.19) | **6.96(1.86-26.31)*** |
| **Objective:Difficulty with expenses (= Yes)** | 0.90(0.72-1.13) | 1.26(0.79-1.99) | 1.55(0.90-2.69) | 0.84(0.64-1.09) | 0.90(0.52-1.58) | 0.83(0.57-1.21) |
| **Objective:Has public health insurance (=Yes)** | 0.79(0.54-1.17) | 0.96(0.28-3.39) | 0.55(0.29-1.07) | 0.90(0.59-1.38) | 0.98(0.39-2.46) | 0.70(0.43-1.16) |
| **Objective:Has private health insurance (=Yes)** | 1.02(0.73-1.43) | 1.51(0.89-2.59) | 1.03(0.69-1.52) | 0.98(0.72-1.34) | 0.61(0.34-1.07) | 1.01(0.50-2.03) |

**Sub Table 6. Interaction effects of modifiable covariates on the association between LGBTQ+ status and unmet mental health needs, among Black populations.** Only the results for interaction were presented. Data were presented as adjusted odds ratios (95% confidence intervals), which were extracted from weighted logistic regression. In the fitted weighted logistic regression, the outcome is the unmet mental health needs (yes or no), and the independent variables include, objective (shown in columns 2-7), age, marital status and its interaction with objective, educational attainment and its interaction with objective, household income and its interaction with objective, difficulty with expenses and its interaction with objective, availability of public health insurance and its interaction with objective, availability of private health insurance and its interaction with objective, and state of residence. “-“ means no corresponding result. * p < 0.05, ** p < 0.01, *** p < 0.001

|  | **Objective** | | | | | |
| --- | --- | --- | --- | --- | --- | --- |
|  | **LGBTQ+** | **Lesbian** | **Gay** | **Bisexual** | **Transgender** | **Queer+** |
| **Marital Status** |  |  |  |  |  |  |
| Objective:Married | Reference | Reference | Reference | Reference | Reference | Reference |
| Objective:Never married | 1.32(0.90-1.93) | 1.32(0.70-2.51) | 0.77(0.32-1.82) | 1.20(0.80-1.79) | 0.44(0.05-3.82) | 1.55(0.73-3.25) |
| Objective:Widowed/divorced/separated | 1.06(0.68-1.65) | 1.22(0.50-3.00) | 0.54(0.18-1.65) | 1.11(0.60-2.03) | 0.98(0.09-10.91) | 1.06(0.48-2.39) |
| **Total household income before taxes** |  |  |  |  |  |  |
| Objective:$0-$34999 | Reference | Reference | Reference | Reference | Reference | Reference |
| Objective:$35,000 - $49,999 | 0.90(0.55-1.43) | 0.83(0.36-1.90) | 0.41(0.11-1.62) | 1.05(0.57-1.95) | 3.19(0.63-16.12) | 0.94(0.49-1.80) |
| Objective:$50,000 - $74,999 | 1.40(0.87-2.27) | 1.72(1.03-2.86). | 0.48(0.20-1.14) | 1.68(0.68-4.10) | 1.57(0.19-13.07) | 1.92(1.02-3.63). |
| Objective:$75,000 - $99,999 | 1.88(0.80-4.44) | 2.05(0.59-7.03) | **0.23(0.07-0.79)*** | **3.00(1.13-8.08)*** | **0.00(0.00-0.00)***** | 2.41(0.79-7.46) |
| Objective:$100000+ | 0.79(0.45-1.39) | 0.99(0.37-2.66) | **0.07(0.02-0.26)***** | 0.94(0.48-1.86) | 2.53(0.25-25.79) | 1.57(0.58-4.22) |
| **Education attained** |  |  |  |  |  |  |
| Objective:Less than high school | Reference | Reference | Reference | Reference | Reference | Reference |
| Objective:Some high school | 0.68(0.18-2.51) | 0.23(0.03-1.82) | 1.90(0.14-26.31) | 0.39(0.06-2.32) | 0.50(0.01-18.36) | 0.32(0.05-1.95) |
| Objective:High school graduate or equivalent | 0.61(0.19-2.03) | **0.11(0.02-0.57)*** | 0.27(0.02-2.92) | 0.92(0.13-6.69) | 0.44(0.01-14.44) | 0.73(0.17-3.25) |
| Objective:Some college, but degree not received or is in progress | 0.66(0.22-1.95) | 0.20(0.04-1.06). | 0.44(0.05-3.71) | 0.79(0.14-4.53) | 1.92(0.16-23.57) | 0.70(0.18-2.83) |
| Objective:Associate’s degree | 0.61(0.15-2.53) | **0.09(0.01-0.58)*** | 0.26(0.04-1.68) | 1.14(0.15-8.85) | 0.09(0.00-4.66) | 0.51(0.09-2.83) |
| Objective:Bachelor's degree | 0.60(0.18-1.97) | 0.18(0.03-0.95). | 0.30(0.03-2.72) | 1.19(0.17-8.41) | 0.10(0.00-4.18) | 0.42(0.08-2.05) |
| Objective:Graduate degree | 0.45(0.14-1.48) | **0.09(0.02-0.54)*** | 0.22(0.03-1.90) | 0.65(0.09-4.71) | 0.46(0.01-15.18) | 0.52(0.10-2.64) |
| **Objective:Difficulty with expenses (= Yes)** | 1.22(0.88-1.70) | 1.01(0.63-1.63) | 1.07(0.58-1.97) | 1.11(0.64-1.90) | 3.10(0.64-15.03) | 1.65(0.94-2.89) |
| **Objective:Has public health insurance (=Yes)** | **0.84(0.72-0.98)*** | 1.34(0.68-2.64) | 0.86(0.41-1.77) | 0.79(0.53-1.19) | 0.66(0.10-4.44) | 0.85(0.46-1.57) |
| **Objective:Has private health insurance (=Yes)** | 0.98(0.73-1.31) | 1.51(0.74-3.03) | 1.67(0.88-3.19) | 0.90(0.61-1.35) | 0.84(0.15-4.81) | 0.84(0.55-1.26) |

**Sub Table 7. Interaction effects of modifiable covariates on the association between LGBTQ+ status and unmet mental health needs, among Hispanic populations.** Only the results for interaction were presented. Data were presented as adjusted odds ratios (95% confidence intervals), which were extracted from weighted logistic regression. In the fitted weighted logistic regression, the outcome is the unmet mental health needs (yes or no), and the independent variables include, objective (shown in columns 2-7), age, marital status and its interaction with objective, educational attainment and its interaction with objective, household income and its interaction with objective, difficulty with expenses and its interaction with objective, availability of public health insurance and its interaction with objective, availability of private health insurance and its interaction with objective, and state of residence. “-“ means no corresponding result. * p < 0.05, ** p < 0.01, *** p < 0.001

|  | **Objective** | | | | | |
| --- | --- | --- | --- | --- | --- | --- |
|  | **LGBTQ+** | **Lesbian** | **Gay** | **Bisexual** | **Transgender** | **Queer+** |
| **Marital Status** |  |  |  |  |  |  |
| Objective:Married | Reference | Reference | Reference | Reference | Reference | Reference |
| Objective:Never married | 1.12(0.85-1.46) | 1.26(0.78-2.01) | 0.99(0.56-1.73) | 0.75(0.50-1.13) | 0.41(0.14-1.19) | 1.49(0.93-2.41) |
| Objective:Widowed/divorced/separated | 1.00(0.75-1.34) | 0.55(0.24-1.27) | 0.77(0.26-2.27) | 0.63(0.35-1.12) | 0.32(0.09-1.16) | 1.35(0.94-1.92) |
| **Total household income before taxes** |  |  |  |  |  |  |
| Objective:$0-$34999 | Reference | Reference | Reference | Reference | Reference | Reference |
| Objective:$35,000 - $49,999 | 1.16(0.75-1.82) | 0.47(0.18-1.23) | 1.36(0.89-2.12) | 1.31(0.76-2.25) | 1.52(0.54-4.26) | 0.97(0.49-1.93) |
| Objective:$50,000 - $74,999 | 1.35(0.84-2.16) | 0.89(0.35-2.23) | 1.42(0.92-2.18) | 1.34(0.59-3.00) | 0.60(0.16-2.29) | 1.46(0.86-2.51) |
| Objective:$75,000 - $99,999 | 0.96(0.70-1.31) | 0.46(0.20-1.07). | 0.80(0.33-1.95) | 1.17(0.80-1.72) | 0.76(0.26-2.25) | 0.79(0.52-1.23) |
| Objective:$100000+ | 1.27(0.87-1.88) | 0.56(0.20-1.54) | 1.52(0.72-3.25) | 1.22(0.70-2.12) | 2.41(0.77-7.61) | 1.34(0.90-1.97) |
| **Education attained** |  |  |  |  |  |  |
| Objective:Less than high school | Reference | Reference | Reference | Reference | Reference | Reference |
| Objective:Some high school | 1.00(0.59-1.70) | 0.47(0.09-2.44) | 0.43(0.07-2.48) | 0.70(0.22-2.18) | 1.02(0.26-4.10) | 1.04(0.58-1.90) |
| Objective:High school graduate or equivalent | 0.69(0.43-1.12) | 1.14(0.26-4.90) | 0.25(0.07-0.90). | 0.43(0.18-1.04). | 0.57(0.13-2.53) | **0.56(0.38-0.83)*** |
| Objective:Some college, but degree not received or is in progress | 1.01(0.66-1.54) | 0.68(0.14-3.46) | 0.75(0.20-2.75) | 0.49(0.20-1.20) | 0.39(0.09-1.67) | 1.02(0.65-1.62) |
| Objective:Associate’s degree | 1.21(0.73-1.97) | 0.79(0.11-5.81) | 0.88(0.28-2.77) | 0.51(0.16-1.67) | 0.76(0.09-6.75) | 1.46(0.90-2.36) |
| Objective:Bachelor's degree | 0.83(0.59-1.17) | 0.42(0.07-2.61) | **0.25(0.07-0.86)*** | 0.41(0.16-1.05). | 0.60(0.18-2.03) | 1.36(0.97-1.93). |
| Objective:Graduate degree | 0.81(0.56-1.19) | 0.51(0.10-2.69) | 0.51(0.16-1.62) | 0.35(0.13-0.97). | 0.55(0.18-1.70) | 1.00(0.63-1.60) |
| **Objective:Difficulty with expenses (= Yes)** | 1.21(1.00-1.46). | **1.73(1.07-2.80)*** | 1.38(1.00-1.92). | **1.65(1.19-2.29)**** | 1.75(0.73-4.18) | 1.16(0.87-1.54) |
| **Objective:Has public health insurance (=Yes)** | 1.20(0.85-1.68) | 0.73(0.28-1.86) | 1.11(0.74-1.63) | 1.22(0.73-2.03) | 1.63(0.73-3.63) | 1.32(0.93-1.90) |
| **Objective:Has private health insurance (=Yes)** | 1.20(0.97-1.48) | 1.58(1.03-2.41). | 1.11(0.70-1.75) | 1.12(0.83-1.51) | 1.42(0.59-3.42) | 1.26(0.97-1.63). |

**Sub Table 8. Association of the risk difference in the unmet mental health needs between LGBTQ+ (or its subtypes) and non-LGBTQ+ with state-level factors relating to the characterizes of mental health facilities.** Because there is a strong correlation between state-level variables, and the number of state-level variables to be fitted is much larger than the number of states, we only include one state-level variable at a time when fitting the data. Only results with significant associations were presented.

| **Objective** | **State-level variable** | **Label of state-level variable** | **Coefficient (95% confidence interval)** |
| --- | --- | --- | --- |
| LGBTQ+ | **a4_2_FACILITYTYPE** | **Separate inpatient psychiatric unit of a general hospital** | **-0.2237(-0.4206--0.0268)** |
| LGBTQ+ | **a3_3_SETTINGDTPH** | **Provides mental health treatment in a partial hospitalization/day treatment sett** | **-0.1408(-0.2343--0.0474)** |
| LGBTQ+ | **a18_18_SRVC32** | **Facility offers dedicated mental health treatment program for persons with HIV o** | **-0.1280(-0.2242--0.0318)** |
| LGBTQ+ | **a1_7_ADMINSERV** | **Facility offers administrative or operational services for mental health treatment** | **-0.1012(-0.1931--0.0093)** |
| LGBTQ+ | **a18_4_SRVC63** | **Facility offers dedicated mental health treatment program for seniors or older a** | **-0.0929(-0.1643--0.0216)** |
| LGBTQ+ | **a14_15_MHVOCREHAB** | **Facility offers vocational rehabilitation services** | **-0.0808(-0.1495--0.0121)** |
| LGBTQ+ | **a13_ANTIPSYCH** | **Antipsychotics for the treatment of serious mental illness (SMI)** | **-0.0661(-0.1175--0.0148)** |
| LGBTQ+ | a30_7_FUNDSTATEWELFARE | Accepts state welfare or child and family services agency funds as source of pay | 0.0533(0.0031-0.1035) |
| LGBTQ+ | a30_15_REVCHK15 | Accepts federal military insurance (such as TRICARE) as source of payment for me | 0.0540(0.0060-0.1021) |
| LGBTQ+ | a28_FEESCALE | Facility uses a sliding fee scale | 0.0580(0.0092-0.1068) |
| LGBTQ+ | a30_13_FUNDCMHG | Accepts Community Mental Health Block Grants as source of payment for mental hea | 0.0586(0.005-0.1123) |
| LGBTQ+ | a30_8_FUNDSTATEJUV | Accepts state corrections/juvenile justice agency funds as source of payment for | 0.0625(0.0184-0.1066) |
| LGBTQ+ | a30_17_REVCHK17 | Accepts IHS/Tribal/Urban (ITU) funds as source of payment for mental health trea | 0.0630(0.0084-0.1176) |
| LGBTQ+ | a30_16_FUNDVA | Accepts U.S. Department of Veterans Affairs funds as source of payment for menta | 0.0674(0.0122-0.1226) |
| LGBTQ+ | a30_2_REVCHK2 | Accepts private health insurance as source of payment for mental health treatmen | 0.0750(0.0015-0.1485) |
| LGBTQ+ | a24_3_QUALREV | Regularly scheduled case review by an appointed quality review committee is part | 0.0854(0.0132-0.1576) |
| LGBTQ+ | a12_14_TREATEMDR | Facility provides Eye Movement Desensitization andReprocessing (EMDR) | 0.0991(0.0461-0.1522) |
| LGBTQ+ | a12_2_TREATFAMTHRPY | Facility offers couples/family therapy | 0.1014(0.0258-0.1771) |
| LGBTQ+ | a30_18_FUNDPRIVCOMM | Accepts Private or Community foundation funds as source of payment for mental he | 0.1035(0.0087-0.1983) |
| LGBTQ+ | a17_1_YNGCHLD | Accepts young children (aged 0-5 years old) for treatment | 0.1098(0.0566-0.1630) |
| LGBTQ+ | a12_9_TREATTRAUMATHRPY | Facility offers trauma therapy | 0.1127(0.0384-0.1870) |
| LGBTQ+ | a17_3_ADOLES | Accepts adolescents (aged 13-17 years old) for treatment | 0.1200(0.0662-0.1738) |
| LGBTQ+ | a17_2_CHILDREN | Accepts children (aged 6-12 years old) for treatment | 0.1281(0.0707-0.1855) |
| LGBTQ+ | a12_15_TREATTELEMEDINCE | Facility offers telemedicine/telehealth therapy | 0.1331(0.0600-0.2062) |
| LGBTQ+ | a30_1_REVCHK1 | Accepts cash or self-payment for mental health treatment services | 0.1400(0.0531-0.2269) |
| LGBTQ+ | a1_1_MHINTAKE | Facility offers mental health intake | 0.1647(0.0287-0.3006) |
| LGBTQ+ | a4_8_FACILITYTYPE | NA | 0.1887(0.0169-0.3605) |
| LGBTQ+ | a12_4_TREATCOGTHRPY | Facility offers cognitive behavioral therapy | 0.1920(0.0312-0.3527) |
| LGBTQ+ | a24_1_CONTED | Continuing education requirements for professional staff is part of facility’s s | 0.2877(0.1035-0.4719) |
| gay | **a1_7_ADMINSERV** | **Facility offers administrative or operational services for mental health treatment** | **-0.2133(-0.4105--0.0161)** |
| gay | **a14_15_MHVOCREHAB** | **Facility offers vocational rehabilitation services** | **-0.1702(-0.3177--0.0228)** |
| gay | **a18_3_SPMI** | **Facility offers dedicated mental health treatment program for persons aged 18 ye** | **-0.1575(-0.3124--0.0025)** |
| gay | **a14_7_ILLNESSMGMT** | **Facility offers illness management and recovery (IMR) services** | **-0.1573(-0.2950--0.0196)** |
| gay | a12_14_TREATEMDR | Facility provides Eye Movement Desensitization and Reprocessing (EMDR) | 0.1776(0.0592-0.2960) |
| bisexual | **a4_2_FACILITYTYPE** | **Separate inpatient psychiatric unit of a general hospital** | **-0.3216(-0.6202--0.0230)** |
| bisexual | **a3_3_SETTINGDTPH** | **Provides mental health treatment in a partial hospitalization/day treatment sett** | **-0.1803(-0.3250--0.0356)** |
| bisexual | **a18_18_SRVC32** | **Facility offers dedicated mental health treatment program for persons with HIV o** | **-0.1786(-0.3252--0.0319)** |
| bisexual | **a14_15_MHVOCREHAB** | **Facility offers vocational rehabilitation services** | **-0.139(-0.2409--0.0370)** |
| bisexual | **a14_29_MHNICOTINEREP** | **Facility offers nicotine replacement therapy** | **-0.1271(-0.2264--0.0277)** |
| bisexual | **a14_30_SMOKINGCESSATION** | **Facility offers non-nicotine smoking/tobacco cessation medications (by prescript** | **-0.1139(-0.2142--0.0135)** |
| bisexual | **a13_ANTIPSYCH** | **Antipsychotics for the treatment of serious mental illness (SMI)** | **-0.0850(-0.1639--0.0060)** |
| bisexual | a28_FEESCALE | Facility uses a sliding fee scale | 0.0811(0.0069-0.1554) |
| bisexual | a30_13_FUNDCMHG | Accepts Community Mental Health Block Grants as source of payment for mental hea | 0.0994(0.0195-0.1793) |
| bisexual | a24_3_QUALREV | Regularly scheduled case review by an appointed quality review committee is part | 0.1120(0.0015-0.2226) |
| bisexual | a30_16_FUNDVA | Accepts U.S. Department of Veterans Affairs funds as source of payment for menta | 0.1214(0.0403-0.2026) |
| bisexual | a17_1_YNGCHLD | Accepts young children (aged 0-5 years old) for treatment | 0.1229(0.0365-0.2093) |
| bisexual | a17_3_ADOLES | Accepts adolescents (aged 13-17 years old) for treatment | 0.1418(0.0543-0.2292) |
| bisexual | a17_2_CHILDREN | Accepts children (aged 6-12 years old) for treatment | 0.1565(0.0640-0.2491) |
| bisexual | a12_15_TREATTELEMEDINCE | Facility offers telemedicine/telehealth therapy | 0.1738(0.0598-0.2879) |
| bisexual | a30_1_REVCHK1 | Accepts cash or self-payment for mental health treatment services | 0.1983(0.0655-0.3312) |
| bisexual | a1_1_MHINTAKE | Facility offers mental health intake | 0.2465(0.0411-0.4518) |
| bisexual | a12_4_TREATCOGTHRPY | Facility offers cognitive behavioral therapy | 0.2513(0.0051-0.4974) |
| bisexual | a24_1_CONTED | Continuing education requirements for professional staff is part of facility’s s | 0.4603(0.1858-0.7348) |
| transgender | a17_1_YNGCHLD | Accepts young children (aged 0-5 years old) for treatment | 0.3608(0.0341-0.6876) |
| transgender | a14_10_FAMPSYCHED | Facility offers family psychoeducation | 0.3995(0.0061-0.7930) |
| transgender | a24_3_QUALREV | Regularly scheduled case review by an appointed quality review committee is part | 0.4523(0.0499-0.8547) |
| transgender | a24_1_CONTED | Continuing education requirements for professional staff is part of facility’s s | 1.3248(0.2725-2.3770) |
| queer | **a12_3_TREATGRPTHRPY** | **Facility offers group therapy** | -0.1098(-0.2123--0.0074) |
| queer | **a21_PSYCHOFF** | **Mobile/off-site psychiatric crisis services** | -0.0693(-0.1351--0.0034) |
| queer | **a13_ANTIPSYCH** | **Antipsychotics for the treatment of serious mental illness (SMI)** | -0.0617(-0.1211--0.0023) |
| queer | **a24_8_RCA** | **Root cause analysis (RCA) is part of facility's standard operating procedures** | -0.0578(-0.1118--0.0039) |
| queer | a12_14_TREATEMDR | Facility provides Eye Movement Desensitization and Reprocessing (EMDR) | 0.0755(0.0109-0.1401) |
| queer | a12_15_TREATTELEMEDINCE | Facility offers telemedicine/telehealth therapy | 0.1104(0.0225-0.1982) |

**Sub Table 9. Association of the risk difference in the unmet mental health needs between LGBTQ+ (or its subtypes) and non-LGBTQ+ with state-level factors relating to the characterizes of mental health facilities, among those aged 18-25.** Because there is a strong correlation between state-level variables, and the number of state-level variables to be fitted is much larger than the number of states, we only include one state-level variable at a time when fitting the data. Only results with significant associations were presented.

| **Objective** | **State-level variable** | **Label of state-level variable** | **Coefficient (95% confidence interval)** |
| --- | --- | --- | --- |
| LGBTQ+ | **a14_15_MHVOCREHAB** | **Facility offers vocational rehabilitation services** | **-0.2133(-0.3787--0.0479)** |
| LGBTQ+ | a17_1_YNGCHLD | Accepts young children (aged 0-5 years old) for treatment | 0.1491(0.0049-0.2933) |
| LGBTQ+ | a30_14_FUNDFEDGRANT | Accepts Federal grants as source of payment for mental health treatment services | 0.2107(0.0260-0.3954) |
| LGBTQ+ | a14_24_MHHIV | Facility provides HIV testing | 0.2532(0.0125-0.4938) |
| LGBTQ+ | a14_6_MHCHRONIC | Facility offers chronic disease/illness management services | 0.2583(0.0371-0.4795) |
| LGBTQ+ | a14_26_MHTB | Facility provides TB screening | 0.2993(0.1310-0.4675) |
| LGBTQ+ | a17_5_ADULT | Accepts adults (aged 26-64 years old) for treatment | 0.3208(0.0411-0.6004) |
| LGBTQ+ | a17_6_SENIORS | Accepts seniors (aged 65 years and older) for treatment | 0.3479(0.1114-0.5844) |
| LGBTQ+ | a14_23_MHHCV | Facility provides testing for Hepatitis C (HCV) | 0.3846(0.1159-0.6533) |
| LGBTQ+ | a14_25_MHSTD | Facility provides STD testing | 0.3876(0.1180-0.6571) |
| LGBTQ+ | a14_22_MHHBV | Facility provides testing for Hepatitis B (HBV) | 0.3978(0.0903-0.7053) |
| LGBTQ+ | a24_1_CONTED | Continuing education requirements for professional staff is part of facility’s s | 0.5704(0.1076-1.0331) |
| lesbian | a22_SIGNLANG | Provides mental health treatment services in sign language for the deaf and hard | 0.3818(0.0227-0.7409) |
| lesbian | a14_20_MHSUICIDE | Facility offers suicide prevention services | 0.4453(0.0224-0.8682) |
| lesbian | a14_30_SMOKINGCESSATION | Facility offers non-nicotine smoking/tobacco cessation medications (by prescript | 0.4478(0.0032-0.8924) |
| lesbian | a29_PAYASST | Facility offers treatment at no charge or minimal payment to clients who cannot | 0.4914(0.0516-0.9313) |
| lesbian | a14_29_MHNICOTINEREP | Facility offers nicotine replacement therapy | 0.5098(0.0695-0.9501) |
| lesbian | a14_9_DIETEXERCOUNSEL | Facility offers diet and exercise counseling | 0.5346(0.1218-0.9474) |
| lesbian | a23_LANG | Facility provides mental health treatment services in a language other than Engl | 0.6200(0.2236-1.0163) |
| lesbian | a14_12_MHHOUSING | Facility offers housing services | 0.6569(0.1490-1.1649) |
| lesbian | a14_6_MHCHRONIC | Facility offers chronic disease/illness management services | 0.7995(0.2076-1.3913) |
| lesbian | a24_6_SATSUR | Periodic client satisfaction surveys are part of facility’s standard operating p | 1.542(0.0245-3.0595) |
| lesbian | a4_6_FACILITYTYPE | Veterans Affairs Medical Center (VAMC) | 2.7968(0.7998-4.7937) |
| gay | **a24_2_CASEREV** | **Regularly scheduled case review with a supervisor is part of facility’s standard** | **-1.8519(-3.1809--0.5230)** |
| gay | **a14_13_SUPPHOUSING** | **Facility offers supported housing programs** | **-0.6004(-1.0533--0.1476)** |
| gay | **a14_1_ASSERTCOMM** | **Facility offers assertive community treatment (ACT)** | **-0.5798(-1.1280--0.0315)** |
| gay | **a14_21_MHCONSUMER** | **Facility offers consumer-run (peer support) services** | **-0.5708(-0.9651--0.1765)** |
| gay | **a14_12_MHHOUSING** | **Facility offers housing services** | **-0.5173(-1.0067--0.0280)** |
| gay | **smoke_allow** |  | **-0.5012(-0.8206--0.1817)** |
| gay | a14_29_MHNICOTINEREP | Facility offers nicotine replacement therapy | 0.4660(0.0491-0.8830) |
| gay | a14_26_MHTB | Facility provides TB screening | 0.5276(0.0667-0.9885) |
| gay | a14_25_MHSTD | Facility provides STD testing | 0.9659(0.2703-1.6616) |
| gay | a14_22_MHHBV | Facility provides testing for Hepatitis B (HBV) | 0.9940(0.2015-1.7866) |
| gay | a26_USEDSECLUSION | Staff have used seclusion or restraint with clients in the 12-month period | 1.1576(0.2651-2.0501) |
| bisexual | **a4_9_FACILITYTYPE** | **Partial hospitalization/day treatment facility** | -0.8406(-1.6703--0.0108) |
| bisexual | **a14_15_MHVOCREHAB** | **Facility offers vocational rehabilitation services** | -0.2444(-0.4683--0.0206) |
| bisexual | a14_26_MHTB | Facility provides TB screening | 0.3165(0.0821-0.5508) |
| bisexual | a14_23_MHHCV | Facility provides testing for Hepatitis C (HCV) | 0.3901(0.0203-0.7600) |
| bisexual | a24_1_CONTED | Continuing education requirements for professional staff is part of facility’s s | 0.8870(0.2857-1.4883) |
| transgender | **a4_11_FACILITYTYPE** | **Multi-setting mental health facility** | **-3.4817(-6.0612--0.9022)** |
| transgender | **a14_17_FOSTERCARE** | **Facility offers therapeutic foster care** | **-1.7891(-3.2721--0.3061)** |
| transgender | **a30_18_FUNDPRIVCOMM** | **Accepts Private or Community foundation funds as source of payment for mental he** | **-0.9182(-1.7508--0.0855)** |
| transgender | **a3_2_SETTINGRC** | **Provides mental health treatment in a 24-hour residential setting** | **-0.7161(-1.4145--0.0177)** |
| transgender | a10_3_OWNERSHP | A public agency or department | 0.4594(0.0559-0.8629) |
| transgender | a20_PSYCHON | Services for psychiatric emergencies onsite | 0.5699(0.0982-1.0416) |
| transgender | a23_LANG | Facility provides mental health treatment services in a language other than Engl | 0.5755(0.0221-1.1288) |
| transgender | a21_PSYCHOFF | Mobile/off-site psychiatric crisis services | 0.6748(0.1768-1.1729) |
| transgender | a1_7_ADMINSERV | Facility offers administrative or operational services for mental health treatme | 0.8401(0.0272-1.6530) |
| transgender | a12_3_TREATGRPTHRPY | Facility offers group therapy | 0.8428(0.0464-1.6391) |
| transgender | a18_18_SRVC32 | Facility offers dedicated mental health treatment program for persons with HIV o | 0.8805(0.0110-1.7500) |
| transgender | a18_8_FIRSTEPPSYCH | Facility offers dedicated mental health treatment program for persons experienci | 0.9003(0.1377-1.6628) |
| transgender | a17_6_SENIORS | Accepts seniors (aged 65 years and older) for treatment | 0.9033(0.0117-1.7948) |
| transgender | a14_6_MHCHRONIC | Facility offers chronic disease/illness management services | 1.1197(0.3397-1.8998) |
| transgender | a18_14_SRVC114 | Facility offers dedicated mental health treatment program for active duty milita | 1.2255(0.0170-2.4341) |
| transgender | a1_3_MHREFERRAL | Facility offers mental health information and/or referral | 1.3405(0.3906-2.2904) |
| transgender | a3_1_SETTINGIP | Provides mental health treatment in a 24-hour hospital inpatient setting | 1.3845(0.3227-2.4463) |
| queer | **a14_17_FOSTERCARE** | **Facility offers therapeutic foster care** | **-0.8324(-1.4332--0.2316)** |
| queer | **a4_4_FACILITYTYPE** | **Residential treatment center for adults** | **-0.3992(-0.7775--0.0209)** |
| queer | **a3_2_SETTINGRC** | **Provides mental health treatment in a 24-hour residential setting** | **-0.3636(-0.6448--0.0824)** |
| queer | **a18_11_SRVC116** | **Facility offers dedicated mental health treatment program for persons who have e** | **-0.2929(-0.5288--0.0570)** |
| queer | **a14_15_MHVOCREHAB** | **Facility offers vocational rehabilitation services** | **-0.2814(-0.5315--0.0314)** |
| queer | **a18_10_POSTTRAUM** | **Facility offers a dedicated mental health treatment program for persons with a d** | **-0.2628(-0.4983--0.0273)** |
| queer | a14_4_MHCOURTORDERED | Facility offers court-ordered outpatient treatment | 0.2605(0.0585-0.4624) |
| queer | a12_15_TREATTELEMEDINCE | Facility offers telemedicine/telehealth therapy | 0.3190(0.0345-0.6035) |
| queer | a14_26_MHTB | Facility provides TB screening | 0.3374(0.0731-0.6018) |
| queer | a1_2_MHDIAGEVAL | Facility offers mental health diagnostic evaluation | 0.4390(0.0119-0.8660) |
| queer | a1_1_MHINTAKE | Facility offers mental health intake | 0.5342(0.0358-1.0327) |
| queer | a17_6_SENIORS | Accepts seniors (aged 65 years and older) for treatment | 0.5582(0.2109-0.9056) |
| queer | a17_5_ADULT | Accepts adults (aged 26-64 years old) for treatment | 0.6540(0.2575-1.0505) |
| queer | a12_13_TREATKIT | Facility provides Ketamine Infusion Therapy (KIT) | 3.2217(0.8313-5.6122) |

**Sub Table 10. Association of the risk difference in the unmet mental health needs between LGBTQ+ (or its subtypes) and Non- LGBTQ+ with state-level factors relating to the characterizes of mental health facilities, among Black populations.** Because there is a strong correlation between state-level variables, and the number of state-level variables to be fitted is much larger than the number of states, we only include one state-level variable at a time when fitting the data. Only reuslts with significant associations were presented.

| **Objective** | **State-level variable** | **Label of state-level variable** | **Coefficient (95% confidence interval)** |
| --- | --- | --- | --- |
| LGBTQ+ | a14_10_FAMPSYCHED | Facility offers family psychoeducation | 0.2752(0.0010-0.5494) |
| LGBTQ+ | a29_PAYASST | Facility offers treatment at no charge or minimal payment to clients who cannot | 0.3180(0.0646-0.5714) |
| LGBTQ+ | a12_15_TREATTELEMEDINCE | Facility offers telemedicine/telehealth therapy | 0.3326(0.0289-0.6362) |
| LGBTQ+ | a14_21_MHCONSUMER | Facility offers consumer-run (peer support) services | 0.3350(0.0893-0.5807) |
| LGBTQ+ | a18_9_SRVC122 | Facility offers dedicated mental health treatment program for persons who have e | 0.4298(0.0075-0.8520) |
| LGBTQ+ | a14_23_MHHCV | Facility provides testing for Hepatitis C (HCV) | 0.4532(0.011-0.8955) |
| LGBTQ+ | a18_7_SPECGRPEATING | Facility offers dedicated mental health treatment program for persons with eatin | 0.6472(0.0856-1.2088) |
| LGBTQ+ | a18_12_TRAUMATICBRAIN | Facility offers dedicated mental health treatment program for persons with traum | 0.8538(0.353-1.3546) |
| LGBTQ+ | a24_2_CASEREV | Regularly scheduled case review with a supervisor is part of facility’s standard | 0.9378(0.0953-1.7802) |
| LGBTQ+ | a12_4_TREATCOGTHRPY | Facility offers cognitive behavioral therapy | 0.9532(0.3573-1.5492) |
| **lesbian** | **a17_4_YOUNGADULTS** | **Accepts young adults (aged 18-25 years old) for treatment** | **-0.9371(-1.8295--0.0447)** |
| **lesbian** | **a23_LANG** | **Facility provides mental health treatment services in a language other than Engl** | **-0.5234(-0.9642--0.0827)** |
| lesbian | a30_5_REVCHK10 | Accepts state-financed health insurance plan other than Medicaid as source of pa | 0.4221(0.0250-0.8191) |
| lesbian | a30_17_REVCHK17 | Accepts IHS/Tribal/Urban (ITU) funds as source of payment for mental health trea | 0.5135(0.1331-0.8939) |
| lesbian | a12_7_TREATBEHAVMOD | Facility offers behavior modification | 0.6197(0.1110-1.1284) |
| lesbian | a1_3_MHREFERRAL | Facility offers mental health information and/or referral | 0.8888(0.1026-1.6749) |
| lesbian | a4_3_FACILITYTYPE | Residential treatment center for children | 1.2632(0.0677-2.4587) |
| lesbian | a14_17_FOSTERCARE | Facility offers therapeutic foster care | 1.6401(0.4653-2.8149) |
| gay | a12_5_TREATDIALTHRPY | Facility offers dialectical behavior therapy | 0.6216(0.0710-1.1722) |
| gay | a30_8_FUNDSTATEJUV | Accepts state corrections/juvenile justice agency funds as source of payment for | 0.6222(0.1757-1.0687) |
| gay | a18_1_SED | Facility offers dedicated mental health treatment program for children/adolescen | 0.6539(0.0836-1.2241) |
| gay | a17_3_ADOLES | Accepts adolescents (aged 13-17 years old) for treatment | 0.738(0.1282-1.3478) |
| gay | a30_17_REVCHK17 | Accepts IHS/Tribal/Urban (ITU) funds as source of payment for mental health trea | 0.7896(0.2546-1.3247) |
| gay | a12_7_TREATBEHAVMOD | Facility offers behavior modification | 0.7968(0.0641-1.5294) |
| gay | a12_9_TREATTRAUMATHRPY | Facility offers trauma therapy | 0.8461(0.0641-1.6281) |
| gay | a12_14_TREATEMDR | Facility provides Eye Movement Desensitization andReprocessing (EMDR) | 0.8496(0.2925-1.4066) |
| gay | a12_4_TREATCOGTHRPY | Facility offers cognitive behavioral therapy | 1.9925(0.3737-3.6113) |
| gay | a4_3_FACILITYTYPE | Residential treatment center for children | 2.3862(0.7440-4.0284) |
| gay | a12_1_TREATPSYCHOTHRPY | Facility offers individual psychotherapy | 2.3965(0.5945-4.1985) |
| **bisexual** | **a12_11_TREATELECTRO** | **Facility offers electroconvulsive therapy** | **-2.6543(-4.9004--0.4083)** |
| bisexual | a18_8_FIRSTEPPSYCH | Facility offers dedicated mental health treatment program for persons experienci | 0.5301(0.0144-1.0457) |
| bisexual | a14_23_MHHCV | Facility provides testing for Hepatitis C (HCV) | 0.6817(0.0080-1.3555) |
| bisexual | a17_5_ADULT | Accepts adults (aged 26-64 years old) for treatment | 0.6838(0.0004-1.3672) |
| bisexual | a18_9_SRVC122 | Facility offers dedicated mental health treatment program for persons who have e | 0.9976(0.3924-1.6029) |
| transgender | a14_16_SUPPEMPLOY | Facility offers supported employment services | 0.7390(0.1123-1.3657) |
| transgender | a14_7_ILLNESSMGMT | Facility offers illness management and recovery (IMR) services | 0.8889(0.1936-1.5842) |
| transgender | a14_21_MHCONSUMER | Facility offers consumer-run (peer support) services | 0.8995(0.1969-1.602) |
| transgender | a14_11_MHEDUCATION | Facility offers education services | 1.0592(0.1936-1.9249) |
| queer | a14_7_ILLNESSMGMT | Facility offers illness management and recovery (IMR) services | 0.2470(0.0359-0.4581) |
| queer | a18_4_SRVC63 | Facility offers dedicated mental health treatment program for seniors or older a | 0.2625(0.0234-0.5016) |
| queer | a18_16_SRVC62 | Facility offers dedicated mental health treatment program for lesbian, gay, bise | 0.3037(0.0373-0.5702) |
| queer | a18_7_SPECGRPEATING | Facility offers dedicated mental health treatment program for persons with eatin | 0.5455(0.0658-1.0251) |
| queer | a4_11_FACILITYTYPE | Multi-setting mental health facility | 1.1103(0.1254-2.0952) |
| queer | a4_6_FACILITYTYPE | Veterans Affairs Medical Center (VAMC) | 1.2709(0.2645-2.2772) |

**Sub Table 11. Association of the risk difference in the unmet mental health needs between LGBTQ+ (or its subtypes) and Non- LGBTQ+ with state-level factors relating to the characterizes of mental health facilities, among Hispanic populations.** Because there is a strong correlation between state-level variables, and the number of state-level variables to be fitted is much larger than the number of states, we only include one state-level variable at a time when fitting the data. Only reuslts with significant associations were presented.

| **Objective** | **State-level variable** | **Label of state-level variable** | **Coefficient (95% confidence interval)** |
| --- | --- | --- | --- |
| LGBTQ+ | **a17_4_YOUNGADULTS** | **Accepts young adults (aged 18-25 years old) for treatment** | -0.3483(-0.6298--0.0669) |
| LGBTQ+ | **a18_18_SRVC32** | **Facility offers dedicated mental health treatment program for persons with HIV o** | -0.2626(-0.4841--0.0411) |
| LGBTQ+ | **a18_16_SRVC62** | **Facility offers dedicated mental health treatment program for lesbian, gay, bise** | -0.2077(-0.3919--0.0235) |
| LGBTQ+ | a28_FEESCALE | Facility uses a sliding fee scale | 0.1182(0.0060-0.2304) |
| LGBTQ+ | a30_15_REVCHK15 | Accepts federal military insurance (such as TRICARE) as source of payment for me | 0.1208(0.0113-0.2302) |
| LGBTQ+ | a30_10_FUNDOTHSTATE | Accepts other state government funds as source of payment for mental health trea | 0.1351(0.0037-0.2665) |
| LGBTQ+ | a30_8_FUNDSTATEJUV | Accepts state corrections/juvenile justice agency funds as source of payment for | 0.1505(0.0513-0.2497) |
| LGBTQ+ | a18_1_SED | Facility offers dedicated mental health treatment program for children/adolescen | 0.1617(0.0349-0.2886) |
| LGBTQ+ | a17_3_ADOLES | Accepts adolescents (aged 13-17 years old) for treatment | 0.1629(0.0254-0.3005) |
| LGBTQ+ | a30_13_FUNDCMHG | Accepts Community Mental Health Block Grants as source of payment for mental hea | 0.1734(0.0557-0.2911) |
| LGBTQ+ | a30_14_FUNDFEDGRANT | Accepts Federal grants as source of payment for mental health treatment services | 0.1848(0.0110-0.3585) |
| LGBTQ+ | a12_7_TREATBEHAVMOD | Facility offers behavior modification | 0.1899(0.0260-0.3537) |
| LGBTQ+ | a14_1_ASSERTCOMM | Facility offers assertive community treatment (ACT) | 0.2060(0.0060-0.4059) |
| LGBTQ+ | a12_9_TREATTRAUMATHRPY | Facility offers trauma therapy | 0.2247(0.0521-0.3972) |
| LGBTQ+ | a12_2_TREATFAMTHRPY | Facility offers couples/family therapy | 0.2247(0.0521-0.3973) |
| LGBTQ+ | a30_16_FUNDVA | Accepts U.S. Department of Veterans Affairs funds as source of payment for menta | 0.2324(0.1173-0.3475) |
| LGBTQ+ | a30_1_REVCHK1 | Accepts cash or self-payment for mental health treatment services | 0.2345(0.0275-0.4415) |
| LGBTQ+ | a12_10_TREATACTVTYTHRPY | Facility offers activity therapy | 0.2392(0.0488-0.4296) |
| LGBTQ+ | a30_17_REVCHK17 | Accepts IHS/Tribal/Urban (ITU) funds as source of payment for mental health trea | 0.2620(0.1550-0.3689) |
| LGBTQ+ | a12_15_TREATTELEMEDINCE | Facility offers telemedicine/telehealth therapy | 0.2963(0.1292-0.4634) |
| LGBTQ+ | a24_5_CQIP | Continuous quality improvement processes is part of facility's standard operatin | 0.3173(0.0462-0.5884) |
| LGBTQ+ | a4_3_FACILITYTYPE | Residential treatment center for children | 0.4484(0.0693-0.8275) |
| LGBTQ+ | a12_4_TREATCOGTHRPY | Facility offers cognitive behavioral therapy | 0.4736(0.1120-0.8352) |
| LGBTQ+ | a14_17_FOSTERCARE | Facility offers therapeutic foster care | 0.4742(0.0921-0.8562) |
| LGBTQ+ | a24_1_CONTED | Continuing education requirements for professional staff is part of facility’s s | 0.6028(0.1779-1.0277) |
| LGBTQ+ | a12_1_TREATPSYCHOTHRPY | Facility offers individual psychotherapy | 0.6857(0.2989-1.0725) |
| **lesbian** | **a3_2_SETTINGRC** | **Provides mental health treatment in a 24-hour residential setting** | -0.6428(-1.2445--0.0411) |
| **lesbian** | **a30_10_FUNDOTHSTATE** | **Accepts other state government funds as source of payment for mental health trea** | -0.4757(-0.9133--0.0381) |
| **lesbian** | **a10_2_OWNERSHP** | **A private non-profit organization** | -0.2808(-0.5322--0.0295) |
| lesbian | a24_8_RCA | Root cause analysis (RCA) is part of facility's standard operating procedures | 0.4044(0.0435-0.7652) |
| lesbian | a19_CRISISTEAM2 | Facility offers crisis intervention team that handles acute mental health issues | 0.4051(0.0508-0.7594) |
| lesbian | a10_3_OWNERSHP | A public agency or department | 0.4566(0.1138-0.7994) |
| lesbian | a22_SIGNLANG | Provides mental health treatment services in sign language for the deaf and hard | 0.4638(0.0534-0.8742) |
| lesbian | a23_LANG | Facility provides mental health treatment services in a language other than Engl | 0.5477(0.0739-1.0216) |
| lesbian | a13_ANTIPSYCH | Antipsychotics for the treatment of serious mental illness (SMI) | 0.5486(0.1632-0.9340) |
| lesbian | a21_PSYCHOFF | Mobile/off-site psychiatric crisis services | 0.5739(0.1423-1.0055) |
| lesbian | a24_4_OUTFUP | Client outcome follow-up after discharge is part of facility’s standard operatin | 0.5747(0.0347-1.1148) |
| lesbian | a14_21_MHCONSUMER | Facility offers consumer-run (peer support) services | 0.5917(0.1004-1.0831) |
| lesbian | a14_10_FAMPSYCHED | Facility offers family psychoeducation | 0.6095(0.0752-1.1438) |
| lesbian | a14_19_MHEMGCY | Facility offers psychiatric emergency walk-in services | 0.6138(0.2326-0.9950) |
| lesbian | a14_20_MHSUICIDE | Facility offers suicide prevention services | 0.6384(0.1650-1.1118) |
| lesbian | a29_PAYASST | Facility offers treatment at no charge or minimal payment to clients who cannot | 0.8165(0.3402-1.2927) |
| lesbian | a12_3_TREATGRPTHRPY | Facility offers group therapy | 0.8440(0.1664-1.5215) |
| lesbian | a20_PSYCHON | Services for psychiatric emergencies onsite | 0.8533(0.4979-1.2087) |
| lesbian | a12_15_TREATTELEMEDINCE | Facility offers telemedicine/telehealth therapy | 0.9107(0.3406-1.4808) |
| lesbian | a18_8_FIRSTEPPSYCH | Facility offers dedicated mental health treatment program for persons experienci | 0.9342(0.2918-1.5765) |
| lesbian | a14_5_MHAOT | Facility provides assisted outpatient treatment (AOT) | 1.2532(0.2465-2.2599) |
| lesbian | a3_1_SETTINGIP | Provides mental health treatment in a 24-hour hospital inpatient setting | 1.2758(0.3666-2.1850) |
| lesbian | a12_1_TREATPSYCHOTHRPY | Facility offers individual psychotherapy | 1.5115(0.1260-2.8970) |
| lesbian | a4_1_FACILITYTYPE | Psychiatric hospital | 1.7417(0.2805-3.2029) |
| lesbian | a4_8_FACILITYTYPE | Certified Community Behavioral Health Clinic (CCBHC) | 1.8409(0.5771-3.1047) |
| lesbian | a4_6_FACILITYTYPE | Veterans Affairs Medical Center (VAMC) | 2.4275(0.0545-4.8004) |
| **gay** | **a30_4_REVCHK5** | **Accepts Medicaid as source of payment for mental health treatment services** | -0.9127(-1.7424--0.0829) |
| gay | a3_3_SETTINGDTPH | Provides mental health treatment in a partial hospitalization/day treatment sett | 0.7651(0.0642-1.4659) |
| gay | a18_5_ALZHDEMENTIA | Facility has a tailored program for persons with Alzheimer’s or dementia | 1.4507(0.1288-2.7725) |
| bisexual | a30_17_REVCHK17 | Accepts IHS/Tribal/Urban (ITU) funds as source of payment for mental health trea | 0.2854(0.0319-0.5389) |
| bisexual | a30_14_FUNDFEDGRANT | Accepts Federal grants as source of payment for mental health treatment services | 0.3737(0.0194-0.7279) |
| bisexual | a12_7_TREATBEHAVMOD | Facility offers behavior modification | 0.3814(0.047-0.7158) |
| bisexual | a12_15_TREATTELEMEDINCE | Facility offers telemedicine/telehealth therapy | 0.4869(0.1314-0.8424) |
| bisexual | a14_25_MHSTD | Facility provides STD testing | 0.6203(0.0943-1.1463) |
| bisexual | a14_22_MHHBV | Facility provides testing for Hepatitis B (HBV) | 0.6513(0.0552-1.2473) |
| bisexual | a14_17_FOSTERCARE | Facility offers therapeutic foster care | 0.7951(0.0009-1.5894) |
| bisexual | a12_4_TREATCOGTHRPY | Facility offers cognitive behavioral therapy | 0.8358(0.0862-1.5854) |
| bisexual | a12_1_TREATPSYCHOTHRPY | Facility offers individual psychotherapy | 1.1856(0.3694-2.0017) |
| bisexual | a4_11_FACILITYTYPE | Multi-setting mental health facility | 1.6555(0.2785-3.0325) |
| transgender | **a18_7_SPECGRPEATING** | **Facility offers dedicated mental health treatment program for persons with eatin** | -1.2693(-2.2975--0.2411) |
| transgender | **a18_14_SRVC114** | **Facility offers dedicated mental health treatment program for active duty milita** | -1.1017(-2.0697--0.1337) |
| transgender | **a18_15_SRVC115** | **Facility offers dedicated mental health treatment program for members of militar** | -1.0668(-1.9206--0.2130) |
| transgender | **a18_18_SRVC32** | **Facility offers dedicated mental health treatment program for persons with HIV o** | -1.0651(-1.7308--0.3994) |
| transgender | **a18_9_SRVC122** | **Facility offers dedicated mental health treatment program for persons who have e** | -1.0110(-1.7685--0.2535) |
| transgender | **a18_16_SRVC62** | **Facility offers dedicated mental health treatment program for lesbian, gay, bise** | -0.5916(-1.1735--0.0098) |
| transgender | a22_SIGNLANG | Provides mental health treatment services in sign language for the deaf and hard | 0.4154(0.0296-0.8011) |
| transgender | a14_27_MHTOBACCOUSE | Facility offers screening for tobacco use | 0.4945(0.0761-0.9129) |
| transgender | a24_3_QUALREV | Regularly scheduled case review by an appointed quality review committee is part | 0.5737(0.0565-1.091) |
| transgender | a14_26_MHTB | Facility provides TB screening | 0.6263(0.1036-1.1490) |
| transgender | a12_10_TREATACTVTYTHRPY | Facility offers activity therapy | 0.7753(0.1820-1.3685) |
| transgender | a12_1_TREATPSYCHOTHRPY | Facility offers individual psychotherapy | 1.4992(0.2106-2.7878) |
| transgender | a24_1_CONTED | Continuing education requirements for professional staff is part of facility’s s | 1.6589(0.3038-3.0139) |
| **queer** | **a18_5_ALZHDEMENTIA** | **Facility has a tailored program for persons with Alzheimer’s or dementia** | -0.6200(-1.2203--0.0198) |
| **queer** | **a18_12_TRAUMATICBRAIN** | **Facility offers dedicated mental health treatment program for persons with traum** | -0.4903(-0.9396--0.0410) |
| **queer** | **a18_15_SRVC115** | **Facility offers dedicated mental health treatment program for members of militar** | -0.4773(-0.8694--0.0851) |
| **queer** | **a17_4_YOUNGADULTS** | **Accepts young adults (aged 18-25 years old) for treatment** | -0.4708(-0.8771--0.0645) |
| **queer** | **a3_1_SETTINGIP** | **Provides mental health treatment in a 24-hour hospital inpatient setting** | -0.4243(-0.8263--0.0222) |
| **queer** | **a18_9_SRVC122** | **Facility offers dedicated mental health treatment program for persons who have e** | -0.4025(-0.7556--0.0494) |
| **queer** | **a18_16_SRVC62** | **Facility offers dedicated mental health treatment program for lesbian, gay, bise** | -0.3446(-0.6040--0.0853) |
| **queer** | **a18_2_TAYOUNGADULTS** | **Facility offers dedicated mental health treatment program for transitional age y** | -0.3402(-0.6259--0.0545) |
| **queer** | **a18_8_FIRSTEPPSYCH** | **Facility offers dedicated mental health treatment program for persons experienci** | -0.3256(-0.6089--0.0423) |
| **queer** | **a23_LANG** | **Facility provides mental health treatment services in a language other than Engl** | -0.3056(-0.5008--0.1105) |
| **queer** | **a18_17_SRVC61** | **Facility offers dedicated mental health treatment program for forensic clients (** | -0.2569(-0.4784--0.0354) |
| **queer** | **a14_10_FAMPSYCHED** | **Facility offers family psychoeducation** | -0.2566(-0.4860--0.0272) |
| **queer** | **a24_8_RCA** | **Root cause analysis (RCA) is part of facility's standard operating procedures** | -0.1662(-0.3215--0.0109) |
| queer | a30_11_FUNDLOCALGOV | Accepts county or local government funds as source of payment for mental health | 0.1454(0.0124-0.2785) |
| queer | a30_8_FUNDSTATEJUV | Accepts state corrections/juvenile justice agency funds as source of payment for | 0.1907(0.0455-0.3358) |
| queer | a30_10_FUNDOTHSTATE | Accepts other state government funds as source of payment for mental health trea | 0.1984(0.0104-0.3864) |
| queer | a3_2_SETTINGRC | Provides mental health treatment in a 24-hour residential setting | 0.2810(0.0236-0.5384) |
| queer | a30_16_FUNDVA | Accepts U.S. Department of Veterans Affairs funds as source of payment for menta | 0.2848(0.1125-0.4571) |
| queer | a30_18_FUNDPRIVCOMM | Accepts Private or Community foundation funds as source of payment for mental he | 0.3350(0.0260-0.644) |
| queer | a30_17_REVCHK17 | Accepts IHS/Tribal/Urban (ITU) funds as source of payment for mental health trea | 0.3574(0.2005-0.5143) |
| queer | a4_3_FACILITYTYPE | Residential treatment center for children | 0.6177(0.0719-1.1636) |
| queer | a14_17_FOSTERCARE | Facility offers therapeutic foster care | 0.6619(0.1123-1.2114) |
| queer | a24_1_CONTED | Continuing education requirements for professional staff is part of facility’s s | 0.6844(0.0566-1.3121) |
